# Genotype‐Dependent Effects of Mechanical Stretch and GATA4‐Targeted Compound 3i‐1262 in Cardiomyopathy Patient–Derived hiPSC‐Cardiomyocytes

**DOI:** 10.1111/bcpt.70284

**Published:** 2026-08-18

**Authors:** Saana Pohjavaara, Sini M. Kinnunen, Heikki Ruskoaho, Katriina Aalto‐Setälä, Mika J. Välimäki, Virpi Talman

**Affiliations:** ^1^ Drug Research Program and Division of Pharmacology and Pharmacotherapy, Faculty of Pharmacy University of Helsinki Helsinki Finland; ^2^ Faculty of Medicine and Health Technology and BioMediTech Institute Tampere University Tampere Finland; ^3^ Heart Hospital Tampere University Hospital Tampere Finland; ^4^ Research Centre for Integrative Physiology and Pharmacology, Institute of Biomedicine, Faculty of Medicine University of Turku Turku Finland

**Keywords:** dilated cardiomyopathy, GATA4, hiPSC‐derived cardiomyocytes, hypertrophic cardiomyopathy, mechanical stretch

## Abstract

Genetic hypertrophic and dilated cardiomyopathies (HCM and DCM, respectively) are characterised by structural and functional abnormalities that can lead to heart failure. However, current therapies mainly reduce symptoms. Patient‐derived human induced pluripotent stem cell (hiPSC)–derived cardiomyocytes provide a valuable platform to study genotype‐specific pathophysiology and pharmacology. We subjected hiPSC‐cardiomyocytes from healthy individuals and from patients carrying pathogenic *MYBPC3* (HCM) or *LMNA* (DCM) mutations to cyclic mechanical stretch, with or without the GATA4‐targeted anti‐hypertrophic compound 3i‐1262, and assessed hypertrophy‐associated, mechanosensitive and metabolism‐related genes by qPCR, and hypertrophy‐related proteins by Western blotting. Compared to control, HCM cardiomyocytes displayed higher basal expression of *NPPB* and *MYH7*, whereas DCM cardiomyocytes exhibited lower basal expression of *NPPB* and *NPPA*. Mechanical stretching induced *NPPB* and *MYH7* upregulation in control cardiomyocytes, delayed *MYH7* upregulation in HCM cardiomyocytes and *NPPA* downregulation in DCM cardiomyocytes. Other mechanosensitive genes, such as *GAL*, *CSRP3* and *SLC16A9*, also exhibited genotype‐ and time‐dependent regulation. In control cardiomyocytes, 3i‐1262 produced limited modulation of stretch‐induced gene expression but showed little or no effect in patient‐derived cardiomyocytes. These findings demonstrate that cardiomyopathy mutations influence gene and protein expression, responses to mechanical stretch and 3i‐1262, underscoring the value of patient‐derived hiPSC‐cardiomyocytes in disease modelling and drug discovery.

## Plain English Summary

Inherited conditions, such as hypertrophic and dilated cardiomyopathies, alter the structure and impair the function of the heart and cause heart failure. To study these diseases, we used heart muscle cells derived from healthy individuals and from patients who have mutations that cause these conditions. Patient cells showed differences in gene and protein expression compared with healthy cells under both normal conditions and stress. They also responded differently to treatment with an experimental chemical compound. These findings highlight that patient‐specific heart muscle cells are a powerful model for studying disease mechanisms and drug effects.

## 1 Introduction and Background

Genetic cardiomyopathies are a heterogeneous group of cardiac diseases caused by pathogenic mutations in genes that lead to structural and functional abnormalities of the myocardium in the absence of increased cardiac load. Hypertrophic cardiomyopathy (HCM) and dilated cardiomyopathy (DCM) are the two most prevalent forms of cardiomyopathy [1]. Both conditions are characterised by cellular pathologies, including cardiomyocyte hypertrophy and apoptosis of cardiomyocytes, myofibrillar disorganisation, myocardial fibrosis and inflammation, which collectively contribute to left ventricular remodelling and the development of cardiac dysfunction [2].

While pharmacological interventions targeting the renin–angiotensin–aldosterone system (RAAS) have demonstrated an ability to mitigate pathological remodelling, current therapeutic approaches for HCM and DCM largely focus on symptom management [1, 3, 4, 5, 6, 7]. These strategies involve, in addition to the RAAS, modulating the sympathetic nervous system and calcium channels [1, 3]; however, they do not directly address the underlying molecular mechanisms of HCM and DCM. Drug development for both HCM and DCM has increasingly focused on directly treating cardiac muscle dysfunction, indicating a shift toward a disease‐specific approach [8, 9]. For example, cardiac myosin inhibitors, such as mavacamten, represent the first disease‐specific treatment for HCM, reducing hypercontractility and improving impaired relaxation [10, 11].

To further develop disease‐modifying strategies, it is crucial to elucidate the pathomechanisms and identify sensitive, specific biomarkers that reflect early phenotypic progression. In this context, patient‐derived human induced pluripotent stem cell (hiPSC)–derived cardiomyocytes provide a valuable platform for investigating the underlying cellular and molecular mechanisms associated with HCM and DCM. They have been established as a robust model for examining cardiomyocyte hypertrophy [12, 13, 14] and the impact of genetic mutations on cardiomyocytes [8, 15], as well as for advancing therapeutic strategy development [14]. Moreover, hiPSC‐cardiomyocytes provide a relevant platform for modelling early‐stage disease, free from confounding alterations associated with long‐standing pathology or prior pharmacological treatment [16].

HCM and DCM are genetically heterogeneous disorders, with pathogenic variants identified in genes encoding diverse classes of proteins, including sarcomeric, cytoskeletal, nuclear envelope, desmosomal and RNA‐binding proteins [8, 15, 17]. Among these, *MYBPC3*, encoding myosin‐binding protein C3, is one of the most frequently mutated genes in HCM, whereas *LMNA*, coding for lamin A/C, is commonly implicated in DCM. For example, the *MYBPC3* truncation mutation Gln1061X [18, 19] and the *LMNA* missense mutation S143P [20] have been identified as pathogenic variants associated with HCM and DCM, respectively. At the cellular level, cardiomyocytes derived from patient‐specific hiPSCs carrying these mutations exhibit distinct phenotypic and functional abnormalities, both under baseline conditions [21, 22, 23] and in response to hypoxic stress [21], when compared with control cardiomyocytes. However, the impact of these mutations on responses to mechanical stimuli, such as cyclic mechanical stretch, has not been studied.

Cardiac transcription factors are central regulators of cardiac development, function and adaptive or maladaptive responses in cardiovascular disease. In particular, the interaction between the key cardiac transcription factors GATA4 and NKX2‐5 is essential for the regulation of genes involved in cardiac development and hypertrophic remodelling [24]. GATA4 and NKX2‐5 physically interact and synergistically activate several cardiac genes, including the natriuretic peptide genes *NPPA* and *NPPB*, which encode natriuretic peptide A (ANP) and natriuretic peptide B (BNP), respectively [24, 25, 26, 27]. Importantly, both transcription factors are required for the full activation of mechanical stretch‐responsive genes such as *NPPA* and *NPPB* [25].

Modulation of cardiac transcription factor networks by small molecules has emerged as a potential therapeutic strategy for cardiac hypertrophy [24, 28, 29]. The small molecule inhibitor 3i‐1000, developed by our group to disrupt the GATA4–NKX2‐5 interaction, attenuates mechanical stretch–induced hypertrophy in neonatal rat ventricular cardiomyocytes, as reflected by reduced cardiomyocyte cell size and decreased *NPPA* and *NPPB* mRNA expression following mechanical stretch [28, 29]. Furthermore, a next‐generation GATA4–NKX2‐5 interaction inhibitor developed in our group, compound 3i‐1262, reduces endothelin‐1–induced pro B‐type natriuretic peptide (proBNP) expression and stretch‐induced *NPPB* mRNA expression in hiPSC‐derived cardiomyocytes [30]. However, the effects of GATA4‐targeted compounds in cardiomyopathy patient–derived cardiomyocytes have not yet been investigated.

Building on our earlier work, which established cyclic stretch as a robust model of pathological hypertrophy, evidenced by increased expression of hypertrophy‐associated genes *NPPA* and *NPPB* [12], we studied here the effects of mechanical stretch on hiPSC‐cardiomyocytes derived from individuals with HCM and DCM. Additionally, we assessed the effects of a GATA4‐targeted small molecule compound, 3i‐1262, both under baseline conditions and in the presence of mechanical load, hypothesising that, given its previously reported ability to modulate gene expression in control hiPSC‐cardiomyocytes [30], the compound may exert similar or enhanced effects in patient‐derived cardiomyocytes. Thus, we investigated here how HCM‐ and DCM‐associated mutations affect cardiomyocyte responses to mechanical stretch and treatment with 3i‐1262.

## 2 Materials and Methods

The study was conducted in accordance with the *Basic & Clinical Pharmacology & Toxicology* policy for experimental and clinical studies [31].

### 2.1 Human‐Induced Pluripotent Stem Cell–Derived Cell Lines

The control cell line iPS(IMR90)‐4 was purchased from WiCell (Madison, WI, USA) [32]. Generation and characterisation of the patient‐derived hiPSC lines have been described earlier [21, 22]. The DCM hiPSCs carry a p.S143P mutation in the *LMNA*, while the HCM hiPSCs carry a Gln1061X mutation in the *MYBPC3*. The cardiomyopathy lines were purchased from the iPS Cells core facility of Tampere University. The presence of the p.S143P mutation (TCC to CCC) in the *LMNA* gene in the DCM hiPSCs and the Gln1061X truncating mutation (CAG to TAG) in the *MYBPC3* gene in the HCM hiPSCs was confirmed by DNA sequencing (Supplementary Information Figure S1) as described in the Supplementary Information [21, 22].

### 2.2 Cell Culture and Differentiation

All reagents used in the cell culture were purchased from Gibco, unless otherwise stated. The culture and differentiation are described in the Supplementary Information.

### 2.3 Cyclic Mechanical Stretching

The cells were subjected to cyclic mechanical stretching using the FX‐5000 Tension System (Flexcell International Corporation) as described earlier [12]. In short, 10%–21% equibiaxial stretch was introduced to cells by vacuum suction at 0.5 Hz for either 24 or 72 h. Control cells from the same differentiation were seeded on the same BioFlex culture plates, but mechanical stretch was not applied. The GATA4‐targeted compound 3i‐1262 [30] at the concentration of 30 μM or vehicle (0.1% DMSO) was added to the cells 1 h before stretching and the treatment was continued throughout the stretching protocol.

### 2.4 RNA Isolation, cDNA Synthesis, Quantitative PCR and Western Blot

RNA isolation, cDNA synthesis, quantitative PCR and Western blot analysis are described in the Supplementary Information [12, 30]. Commercial TaqMan Gene Expression Assays (Thermo Fisher Scientific) are listed in Supplementary Information Table S2.

### 2.5 Statistical Analysis

Each treatment group consisted of three to nine independent experiments of cells from individual differentiations. For qPCR data analysis, the average of the technical replicates was used to represent one biological replicate (*n* = 1). Results are expressed as mean with error bars representing the SEM, and dots representing individual independent experiments. Because the normality of the qPCR data could not be reliably assessed, pairwise comparisons were performed using the nonparametric Mann–Whitney *U* test. For Western blot analyses, equality of variances was assessed using Levene's test, and Welch's *t*‐test was applied when unequal variances were detected, as it is appropriate for two‐group comparisons when variances differ. A *p*‐value of <0.05 was considered statistically significant. All statistical analyses were performed in IBM SPSS Statistics 30.0.0.0.

## 3 Results

### 3.1 Hypertrophy‐Associated Gene Expression at Baseline and in Response to Mechanical Stretch and 3i‐1262 Treatment

**FIGURE 1:**
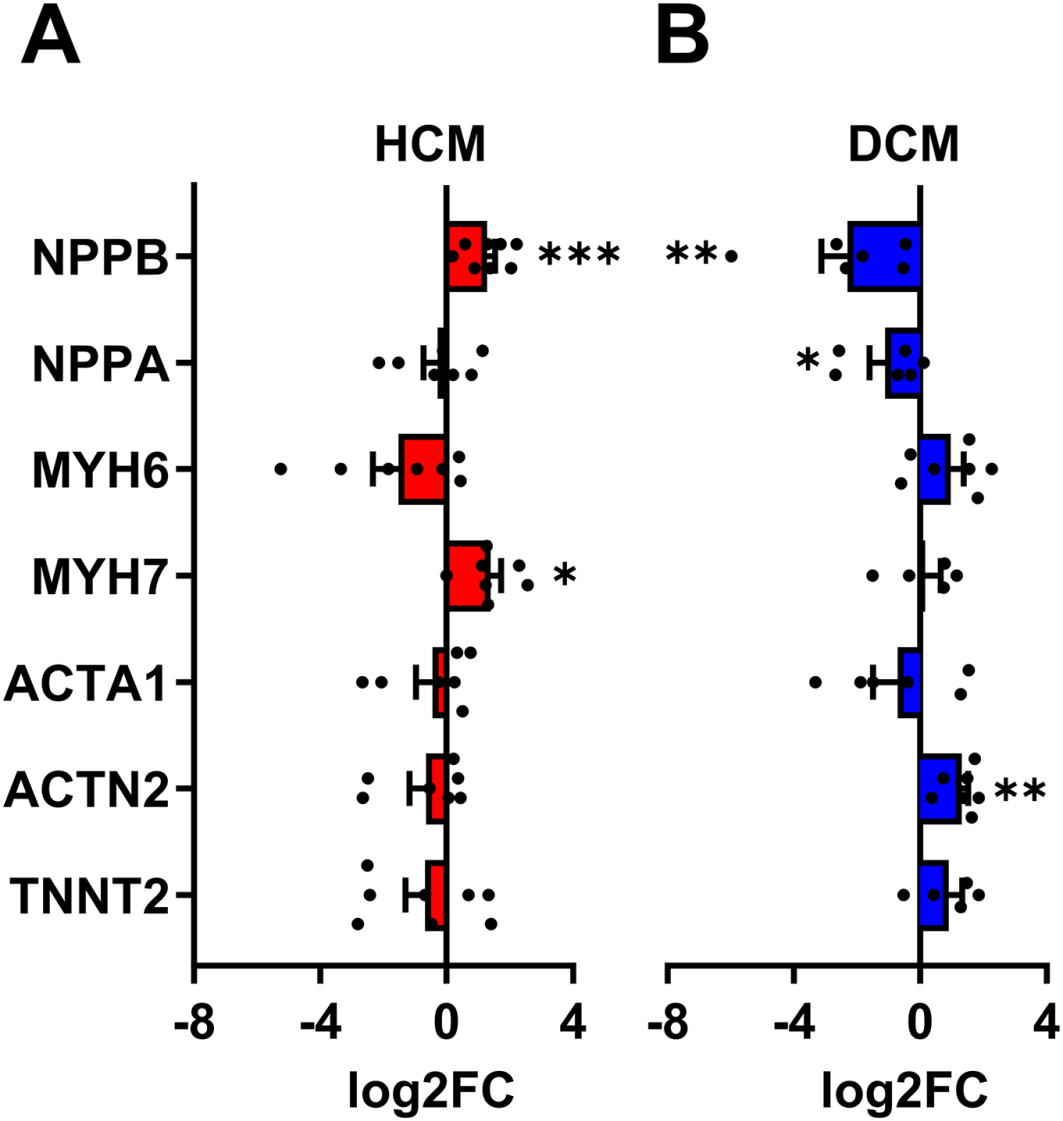
Effects of cardiomyopathy‐associated mutations on the expression of hypertrophy‐related genes. Baseline gene expression of HCM (A) and DCM (B) cardiomyocytes. The bars represent the mean, error bars represent SEM, and dots represent results from individual independent experiments. Results are normalised to the control line. *n* = 5–7, *p < 0.05; **p < 0.01; ***p < 0.001 (Mann–Whitney *U* test). log2FC, log2 fold change.

To investigate baseline differences in hypertrophy‐associated genes between cardiomyopathies, we analysed the expression of a set of key genes in DCM and HCM cardiomyocytes using qPCR [33, 34]. In HCM cardiomyocytes, *NPPB*, which encodes natriuretic peptide B, and *MYH7*, which encodes the β‐myosin heavy chain (β‐MHC) isoform, were upregulated (Figure 1A). In contrast, DCM cardiomyocytes exhibited downregulation of *NPPB* and *NPPA* (coding for atrial natriuretic peptide), whereas *ACTN2*, which encodes alpha‐actinin‐2, was upregulated (Figure 1B).

**FIGURE 2:**
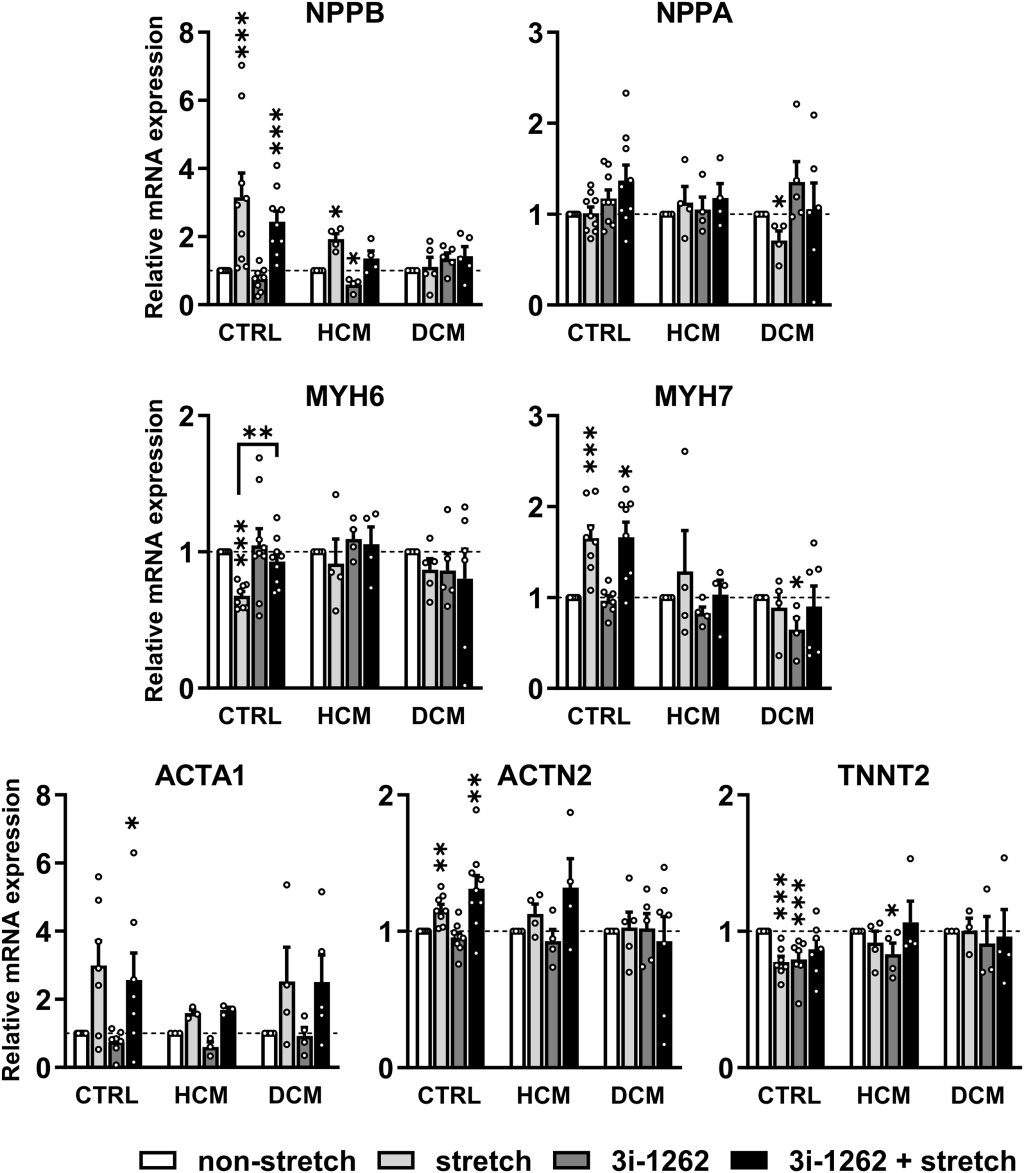
Differential gene expression in hiPSC‐cardiomyocytes after 24 h of mechanical stretching, 3i‐1262 treatment or their combination. Results are presented as mean + SEM and normalised to the respective unstretched sample. *n* = 3–9, *p < 0.05; **p < 0.01; ***p < 0.001 compared to the respective unstretched sample, or as indicated (Mann–Whitney *U* test). CTRL, control cardiomyocytes; HCM, HCM cardiomyocytes; DCM, DCM cardiomyocytes.

To investigate stretch‐induced changes in gene expression, we subjected cardiomyocytes to mechanical stretching [12]. As expected, control cardiomyocytes showed upregulation of the hypertrophic markers *NPPB* and *MYH7* after 24 h of stretch (Figure 2). This was accompanied by downregulation of *MYH6*, which encodes the α‐myosin heavy chain (α‐MHC) isoform. Mechanical stretching also altered the expression of other genes, including upregulation of *ACTN2* and downregulation of *TNNT2*, which encodes the cardiac muscle isoform of troponin T (cTnT). *NPPB* was also upregulated in HCM cardiomyocytes following 24 h of stretching; however, the upregulation was less pronounced than in control cardiomyocytes. In contrast, no significant changes in *NPPB* expression were detected in DCM cardiomyocytes, and the hypertrophy marker *NPPA* was downregulated.

The GATA4‐targeted compound 3i‐1262 has been shown to modulate hypertrophic gene expression in hiPSC‐cardiomyocytes [30]. We therefore evaluated the effect of 3i‐1262 treatment on the gene expression patterns of control, HCM and DCM cardiomyocytes. A 24‐h exposure to 3i‐1262 at 30 μM reduced *NPPB* expression in HCM cardiomyocytes, exhibited a not significant 25% decrease in control cardiomyocytes (*p* = 0.0503), but did not affect *NPPB* expression in DCM cardiomyocytes (Figure 2). Additionally, 3i‐1262 altered sarcomeric gene expression across all lines: it downregulated *MYH7* in DCM cardiomyocytes and *TNNT2* in control and HCM cardiomyocytes.

Compound 3i‐1262 has previously been reported to inhibit stretch‐induced upregulation of *NPPB* expression in control hiPSC‐cardiomyocytes [30]. To determine whether 3i‐1262 modulates stretch responses in DCM and HCM cells, cardiomyocytes were treated with 3i‐1262 at 30 μM. In control cardiomyocytes, exposure to 3i‐1262 for 24 h attenuated the stretch‐induced downregulation of *MYH6*. In addition, 3i‐1262 tended to attenuate the stretch‐induced increase in *NPPB* in both control and HCM cardiomyocytes, reducing the response from 3.2‐fold to 2.4‐fold (*p* = 0.730) and from 1.9‐fold to 1.4‐fold (*p* = 0.114), respectively. Moreover, certain changes in gene expression relative to the corresponding unstretched controls were observed only when cells were subjected to both 3i‐1262 and mechanical stretch. Notably, *NPPB*, *MYH7*, skeletal muscle α‐actin (*ACTA1*) and *ACTN2* were upregulated in control cardiomyocytes under these conditions (Figure 2). However, these changes were comparable to those induced by mechanical stretch alone, indicating that 3i‐1262 does not affect stretch‐induced upregulation of these hypertrophy‐associated genes.

**FIGURE 3:**
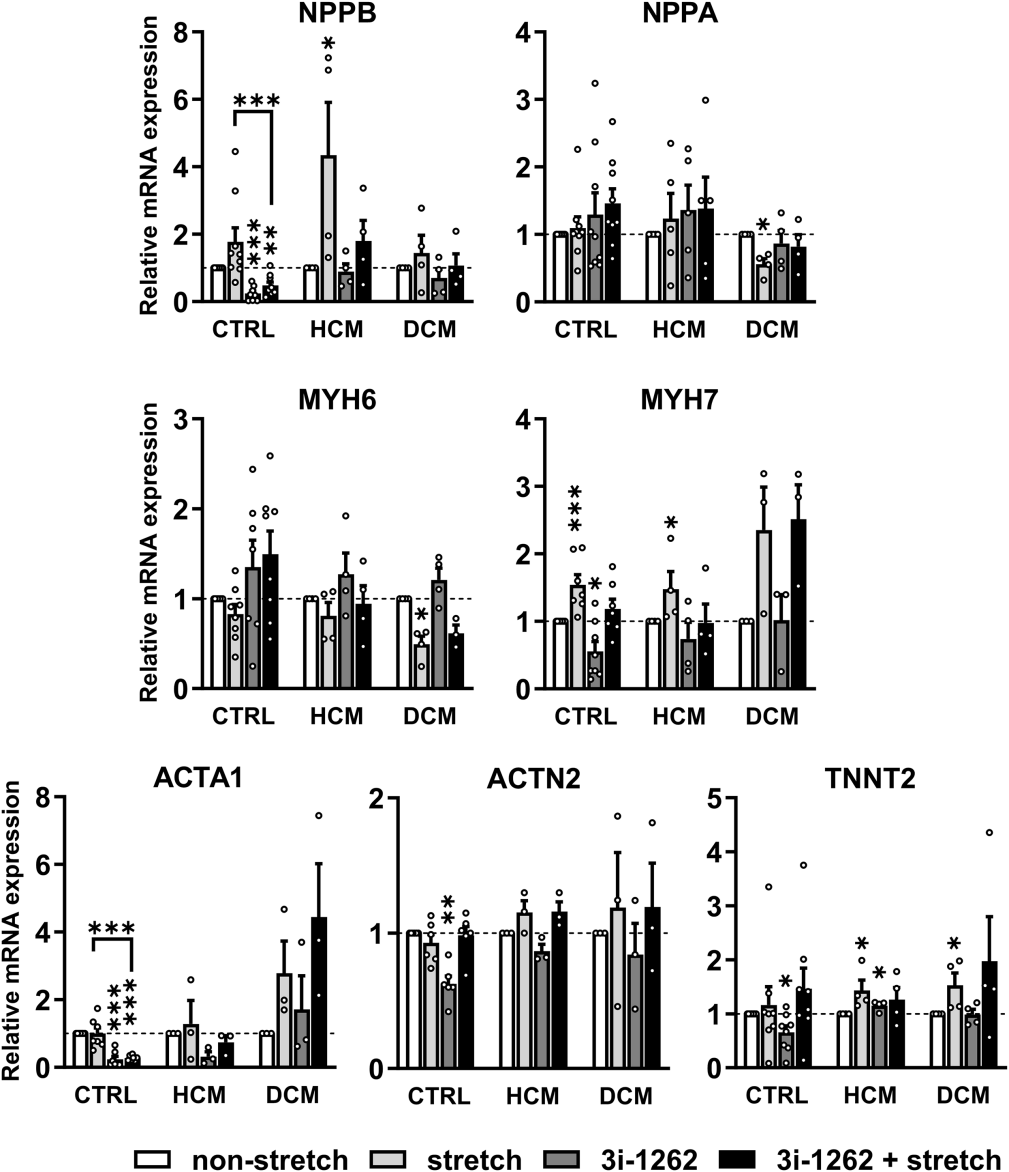
Differential gene expression in hiPSC‐cardiomyocytes induced by 72 h of mechanical stretching, 3i‐1262 treatment or their combination. Results are presented as mean + SEM and normalised to the respective unstretched sample. *n* = 3–9, *p < 0.05; **p < 0.01; ***p < 0.001 compared to the respective unstretched sample, or as indicated (Mann–Whitney *U* test). CTRL, control cardiomyocytes; HCM, HCM cardiomyocytes; DCM, DCM cardiomyocytes.

As mechanical stretching for 72 h is known to induce distinct gene expression profiles in hiPSC‐cardiomyocytes compared to 24‐h stretching [12], we next examined responses of patient‐derived hiPSC‐cardiomyocytes to 72 h of stretching. *NPPB* expression remained slightly, although not significantly elevated, in control cardiomyocytes (1.8‐fold, *p* = 0.0503; Figure 3), being less pronounced than at 24 h. *NPPB* expression remained upregulated also in HCM cardiomyocytes (Figure 3). Notably, *NPPB* upregulation tended to be more pronounced in HCM cardiomyocytes than in control cardiomyocytes (*p* = 0.106), suggesting a shift from the trend observed following 24 h of stretch. Stretching for 72 h also maintained increased *MYH7* gene expression in control cardiomyocytes and induced *MYH7* upregulation in HCM cardiomyocytes. Similarly, *NPPA* expression remained downregulated in DCM cardiomyocytes. Prolonged stretching for 72 h also induced downregulation of *MYH6* and upregulation of *TNNT2* in DCM cardiomyocytes.

Following 72 h of 3i‐1262 exposure, distinct transcriptional responses were observed (Figure 3). The expression of *NPPB*, *MYH7*, *ACTA1*, *ACTN2* and *TNNT2* was downregulated in control cardiomyocytes compared to vehicle controls. In HCM cardiomyocytes, among these genes, *TNNT2* was upregulated following treatment, whereas other genes remained unchanged. Interestingly, 3i‐1262 did not significantly alter any of the hypertrophy‐associated genes examined in DCM cardiomyocytes.

The slight but not significant stretch‐induced increase of *NPPB* in control cardiomyocytes was attenuated by 3i‐1262 at 72 h (Figure 3). The compound 3i‐1262 did not affect the stretch‐induced increases of *MYH7* expression in control and HCM cardiomyocytes, or *NPPB* upregulation in HCM cardiomyocytes. Notably, although *ACTA1* expression in control cardiomyocytes was not changed upon the 72‐h stretch, it was downregulated when combined with 3i‐1262; however, this effect is likely due to the compound.

Taken together, these results suggest that mechanical stretch and 3i‐1262 modulate hypertrophy‐associated gene expression in hiPSC‐cardiomyocytes in a time‐ and genotype‐dependent manner, with control cardiomyocytes exhibiting more pronounced hypertrophic responses, while patient‐derived cells display delayed or blunted responses.

### 3.2 Effects of Cardiomyopathy‐Associated Mutations, Mechanical Stretch and 3i‐1262 on Hypertrophy‐Associated Protein Expression

**FIGURE 4:**
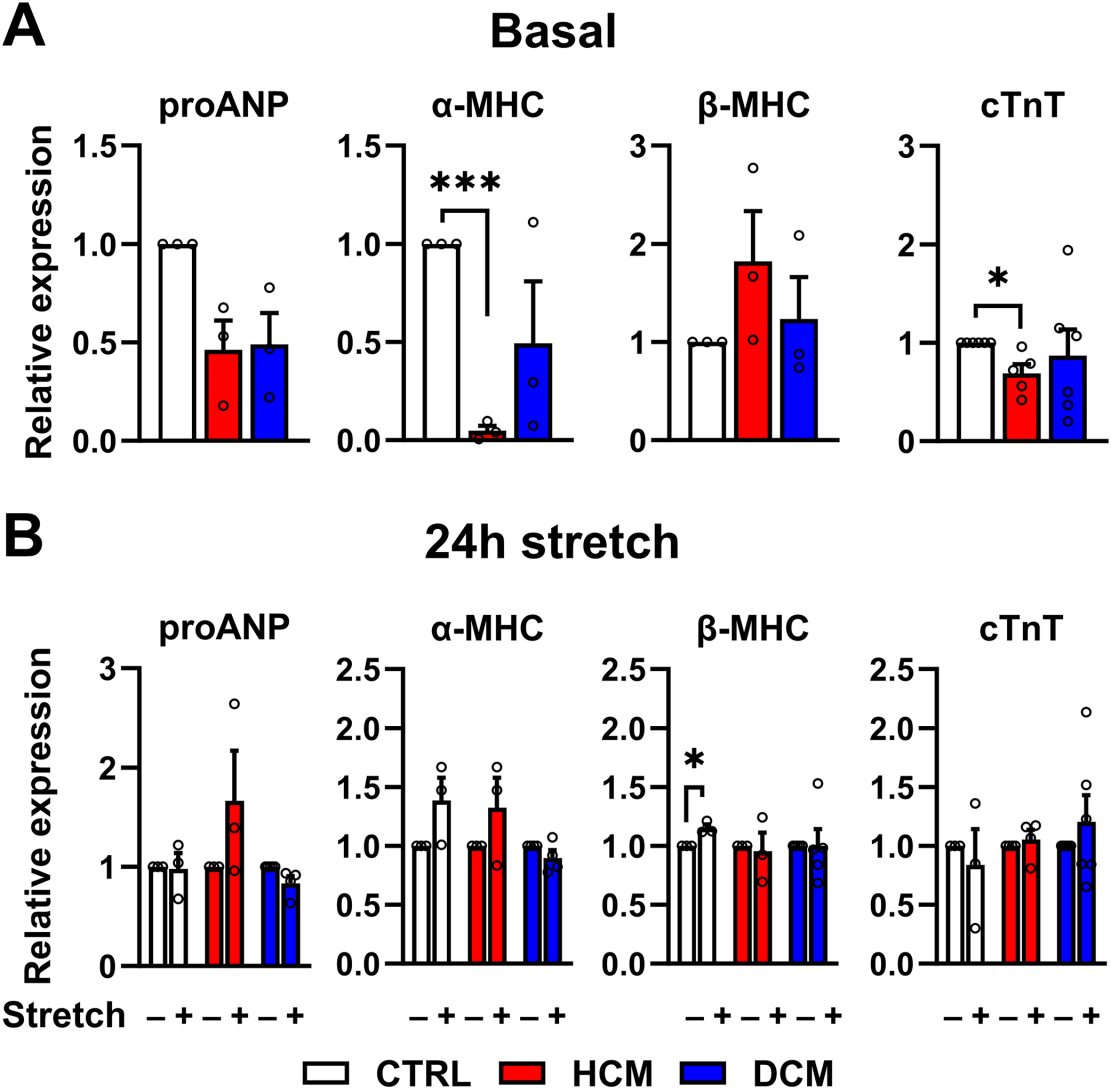
Quantification of proANP, α‐MHC, β‐MHC and cTnT protein expression in control (CTRL), HCM, and DCM hiPSC‐cardiomyocytes. (A) Basal protein levels are presented as mean + SEM and normalised to the control line (*n* = 3–6; *p < 0.05; ***p < 0.001; Welch's *t*‐test). (B) Effects of 24 h mechanical stretch on protein levels. Results are presented as mean + SEM and normalised to the respective, unstretched sample (*n* = 3–6; *p < 0.05; Welch's *t*‐test). See Supplementary Information Figures S2–S5 for the original Western blots and corresponding total protein images.

We next investigated whether the observed changes in gene expression corresponded to changes in protein levels. Although *NPPB* expression was upregulated in control and HCM cardiomyocytes following mechanical stretch, the expression of proBNP, the intracellular prohormone encoded by *NPPB*, did not increase to detectable levels under either basal or stretched conditions. In contrast, proBNP was readily detected following endothelin‐1 treatment, which served as a positive control (Supplementary Information Figures S2–S5) [30]. Under basal conditions, pro‐natriuretic peptide A (proANP), encoded by *NPPA*, tended to be reduced by 54% in HCM (*p* = 0.068) and by 52% in DCM cardiomyocytes (*p* = 0.087; Figure 4A). Notably, α‐MHC and cardiac troponin T (cTnT), encoded by *MYH6* and *TNNT2*, respectively, were lower in HCM cardiomyocytes.

Following 24 h of mechanical stretch, proANP expression tended to increase 1.7‐fold in HCM cardiomyocytes (*p* = 0.318) and decrease 17% in DCM cardiomyocytes (*p* = 0.098; Figure 4B). β‐MHC, encoded by *MYH7*, was upregulated in control cardiomyocytes, whereas no changes were observed in the patient‐derived cell lines. At 72 h, no changes were detected in protein levels in response to stretching, 3i‐1262 treatment or their combination (Supplementary Information Figures S6 and S7).

### 3.3 Expression of Mechanosensitive and Metabolism‐Related Genes Under Baseline Conditions, Mechanical Stretch and 3i‐1262 Treatment

Given that cardiomyopathy‐causing mutations, mechanical stretching and 3i‐1262 induced changes in genes linked to hypertrophy and maturation, we expanded the gene panel. *ACTC1* and *MYBPC3* are among the key sarcomeric and sarcomere‐regulatory genes, coding for actin alpha cardiac muscle 1 and myosin‐binding protein C3, respectively [35], and *GATA4*, a master cardiac transcription factor, is known to regulate stretch‐responsive genes [24]. Additionally, we selected three genes (*CSRP3*, *GAL* and *SLC16A9*), encoding cysteine‐ and glycine‐rich protein 3, galanin and solute carrier family 16 member 9, respectively, which we recently identified as highly stretch‐responsive genes in control hiPSC‐cardiomyocytes [12].

**FIGURE 5:**
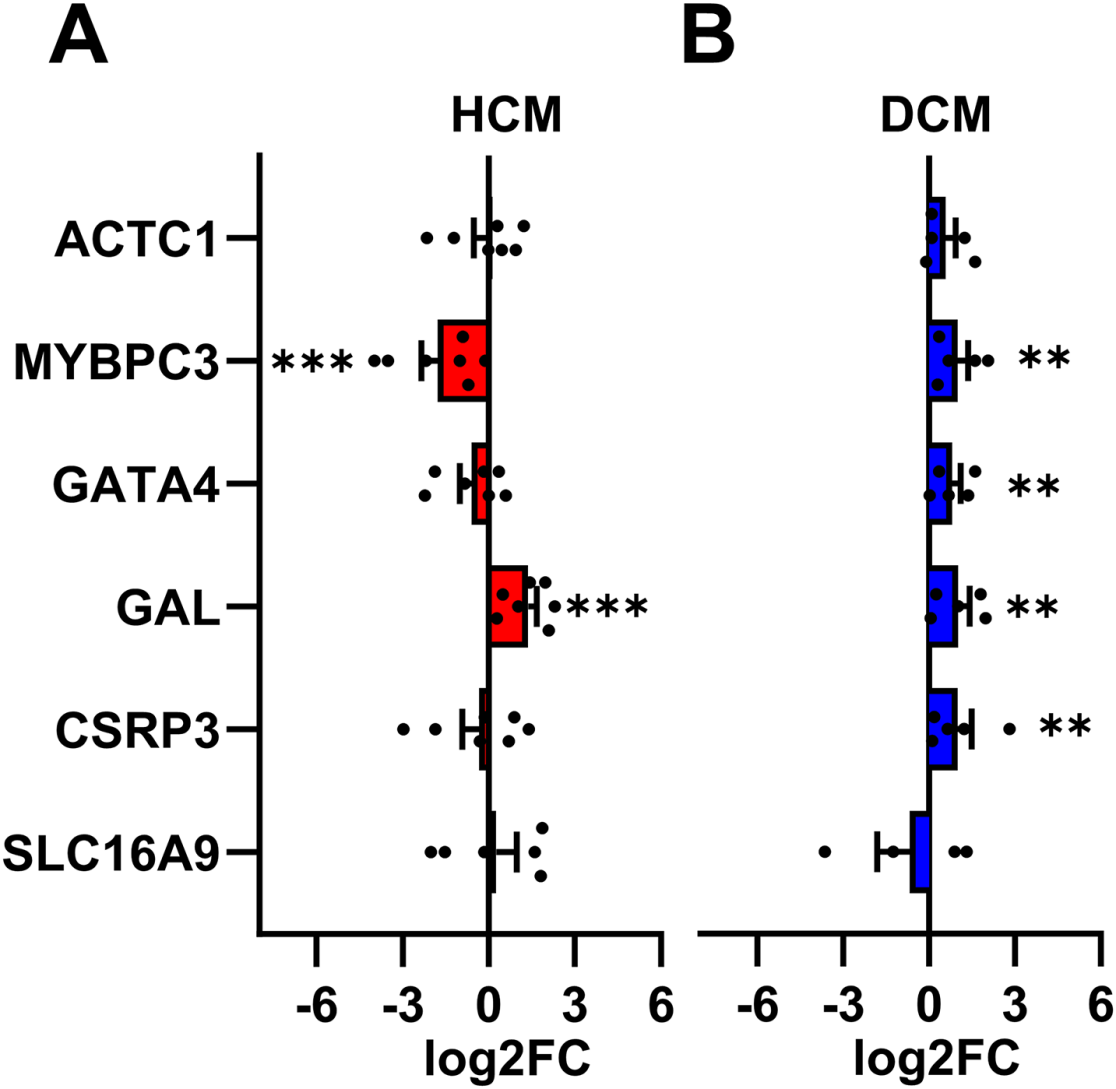
Effects of the cardiomyopathy‐causing mutations on the expression of mechanosensitive and metabolism‐related genes. Baseline gene expression of HCM (A) and DCM (B) cardiomyocytes. The bars represent the mean, error bars represent SEM and dots represent results from individual independent experiments. Results are normalised to the control line. *n* = 5–7, **p < 0.01; ***p < 0.001 (Mann–Whitney *U* test). log2FC, log2 fold change.

At baseline, *GAL* expression was higher in both HCM and DCM cardiomyocytes (Figure 5). In HCM cardiomyocytes, *MYBPC3* expression was lower, as expected, due to the Gln1061X truncating mutation (CAG to TAG) in one allele of the *MYBPC3* gene, which introduces a premature stop codon, prohibiting the detection of mRNA produced from the mutated allele with the TaqMan gene expression assay used in this study. In contrast, *MYBPC3* expression was higher in DCM cardiomyocytes. Additionally, both *CSRP3* and *GATA4* showed higher baseline expression in DCM cardiomyocytes.

#### 3.3.1 Mechanosensitive Gene Expression

**FIGURE 6:**
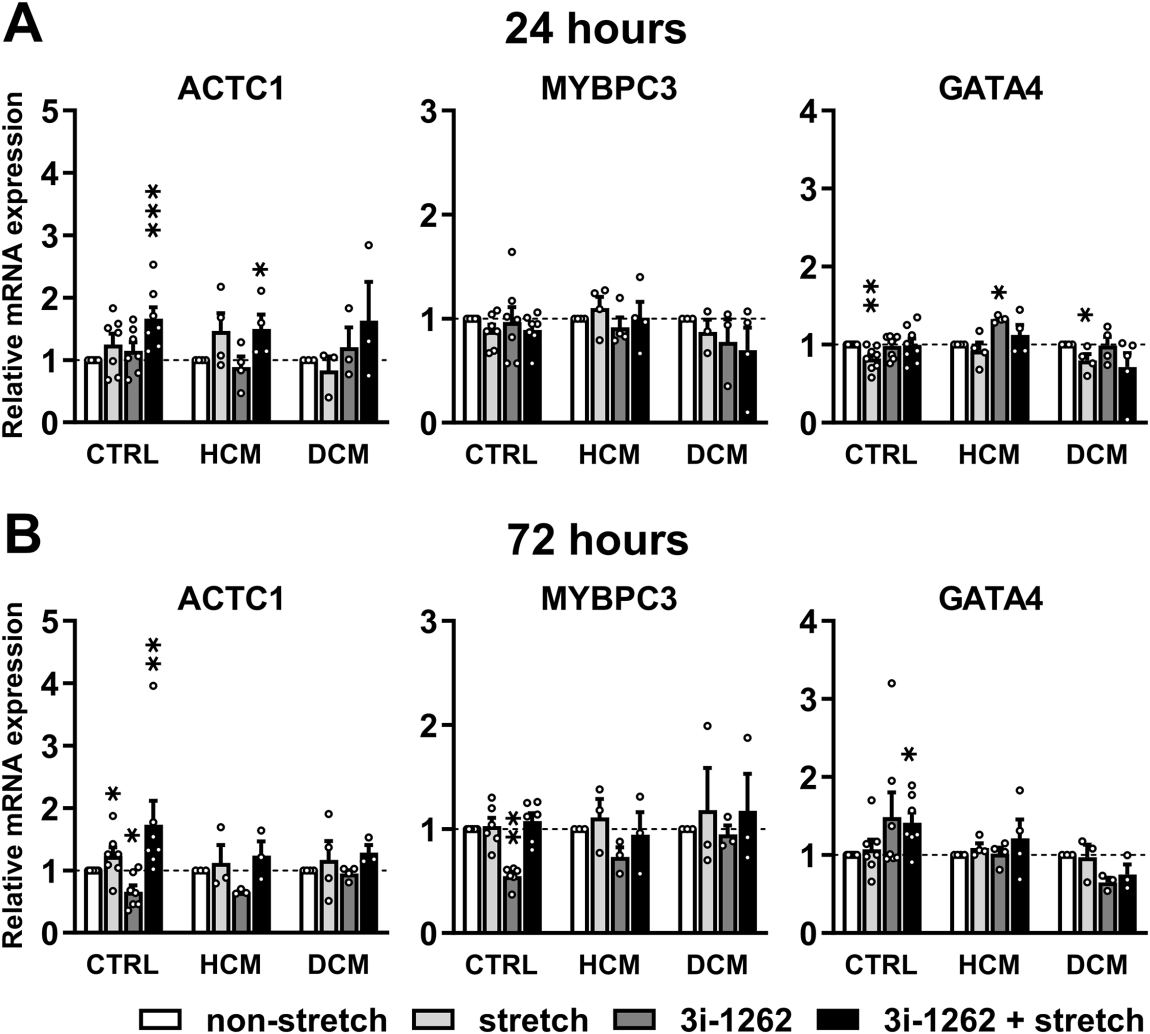
Mechanosensitive gene expression in hiPSC‐cardiomyocytes following 24 or 72 h of mechanical stretch, 3i‐1262 treatment or their combination. Results are presented as mean + SEM and normalised to the respective unstretched sample. *n* = 3–8, *p < 0.05; **p < 0.01; ***p < 0.001 compared to the respective unstretched sample (Mann–Whitney *U* test). CTRL, control cardiomyocytes; HCM, HCM cardiomyocytes; DCM, DCM cardiomyocytes.

After 24 h of mechanical stretch, the cardiac transcription factor *GATA4* was downregulated in both control and DCM cardiomyocytes (Figure 6A). Treatment with 3i‐1262 increased *GATA4* expression in HCM cardiomyocytes (Figure 6A). In the presence of 3i‐1262, stretch induced upregulation of *ACTC1* in control and HCM cardiomyocytes compared with unstretched samples (Figure 6A). After 72 h of stretching, *ACTC1* was upregulated in control cardiomyocytes (Figure 6B). In contrast, following 72 h of 3i‐1262 treatment, both *ACTC1* and *MYBPC3* were downregulated in control cardiomyocytes (Figure 6B). Combined treatment with 3i‐1262 and stretch for 72 h sustained upregulation of *ACTC1* in control cardiomyocytes, whereas HCM cells no longer showed any changes (Figure 6B). Additionally, *GATA4* expression was increased in control cardiomyocytes under the same conditions.

#### 3.3.2 Metabolism‐Related Gene Expression

**FIGURE 7:**
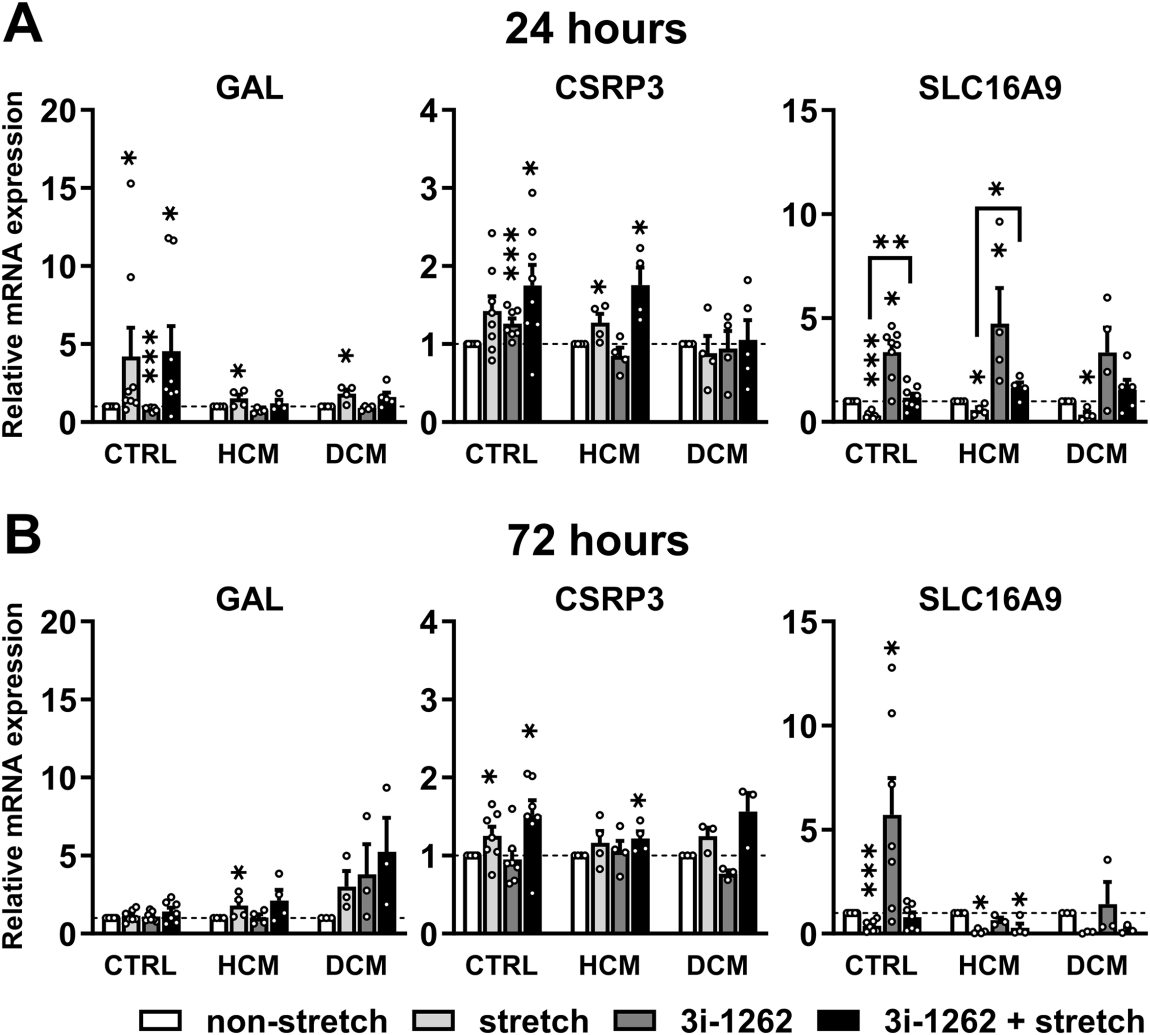
Metabolism‐related gene expression in hiPSC‐cardiomyocytes following 24 h (A) or 72 h (B) of mechanical stretch, 3i‐1262 treatment or their combination. Results are presented as mean + SEM and normalised to the respective unstretched sample. *n* = 3–7, *p < 0.05; **p < 0.01; ***p < 0.001 compared to the respective unstretched sample, or as indicated (Mann–Whitney *U* test). CTRL, control cardiomyocytes; HCM, HCM cardiomyocytes; DCM, DCM cardiomyocytes.

Among the metabolism‐related genes, *GAL* was upregulated and *SLC16A9* was downregulated in all cell lines after 24 h of stretching (Figure 7A), whereas CSRP3 was upregulated only in HCM cardiomyocytes. A 24‐h treatment with 3i‐1262 downregulated *GAL* and upregulated *CSRP3* in control cardiomyocytes and induced upregulation of *SLC16A9* in control and HCM cells, with a similar trend in DCM cells (3.3‐fold, *p* = 0.343). Combined treatment with 3i‐1262 and stretch for 24 h inhibited the stretch‐induced downregulation of *SLC16A9* in control and HCM cardiomyocytes (Figure 7A). Additionally, *GAL* and *CSRP3* were upregulated in control cardiomyocytes relative to unstretched controls, and *CSRP3* was similarly upregulated in HCM cardiomyocytes.

After 72 h of stretching, *GAL* expression increased in HCM cardiomyocytes, which is consistent with the 24‐h response. In DCM cardiomyocytes, *GAL* expression also tended to increase (3‐fold, *p* = 0.1), and no changes were observed in control cells (Figure 7B). *CSRP3* was upregulated in control cardiomyocytes, whereas *SLC16A9* remained downregulated in control and HCM cardiomyocytes. A similar, nonsignificant trend was observed in DCM cardiomyocytes (94% decrease, *p* = 0.1). After 72 h of 3i‐1262 treatment, *SLC16A9* remained upregulated in control cardiomyocytes, whereas HCM and DCM cells showed no significant change. The expression of the other metabolism‐related genes, *CSRP3* and *GAL*, remained unchanged across all cell lines (Figure 7B). Combined exposure to 3i‐1262 and mechanical stretch induced changes similar to those observed at 24 h (Figure 7B), with *CSRP3* upregulated in control and HCM cardiomyocytes. Moreover, the stretch‐counteracting effect of 3i‐1262 on *SLC16A9* expression observed at 24 h in control and HCM cells was no longer evident at 72 h.

Overall, these results demonstrate that mechanical stretch and 3i‐1262 influence mechanosensitive and metabolism‐related gene expression in hiPSC‐cardiomyocytes, with responses varying according to genotype and exposure duration: while control cardiomyocytes exhibit more pronounced transcriptional changes, HCM and DCM cardiomyocytes display attenuated or altered responses.

## 4 Discussion

Consistent with the known role of genetic mutations in cardiomyopathies, *MYBPC3* and *LMNA* variants are among the most common drivers of HCM and DCM, respectively [1]. In cardiomyocytes, these mutations contribute to diverse pathological phenotypes, including upregulation of hypertrophy‐associated genes such as *NPPB*, *NPPA* and *MYH7*; sarcomeric disorganisation and increased susceptibility to arrhythmic events, among other pathological changes [1, 21, 22, 34]. Together, these observations suggest that genotype‐specific effects influence cardiomyocyte phenotypes and may contribute to the variability observed in hypertrophy, mechanosensitivity and responses to pharmacological interventions in vitro. In this study, we investigated how the *MYBPC3* Gln1061X mutation and the *LMNA* S143P mutation influence hypertrophy‐associated gene expression in patient‐derived hiPSC‐cardiomyocytes. In HCM cardiomyocytes, *NPPB* and *MYH7* were upregulated at baseline, consistent with a hypertrophied phenotype observed in previous studies [22]. However, in DCM cardiomyocytes, both *NPPB* and *NPPA* were downregulated. This is in contrast with previous studies using DCM patient–derived hiPSC‐cardiomyocytes carrying other disease‐causing mutations, which generally report upregulation of *NPPA* and *NPPB* along with other hypertrophy‐associated genes [34, 36, 37]. To our knowledge, our study is the first to report baseline downregulation of *NPPA* and *NPPB* in hiPSC‐cardiomyocytes carrying a *LMNA* mutation.

The cardiac hormones natriuretic peptides A and B, encoded by *NPPA* and *NPPB* genes, are released in response to stretch and regulate blood pressure and volume, cardiac hypertrophy, renal function and vasodilatation [38, 39]. Because these genes are well‐established mechanosensitive markers in cardiomyocytes, their unexpected downregulation in DCM cardiomyocytes suggests an altered response to mechanical stretching and a potential defect in mechanotransduction. Mechanotransduction is a process by which cells convert mechanical stimuli from the extracellular environment or from intrinsic cell‐generated forces into biochemical signals, and it regulates downstream responses, such as gene expression [40, 41]. Moreover, mechanotransduction signalling is essential for cardiac adaptation under stress [41]. Even under baseline conditions, spontaneously beating hiPSC‐cardiomyocytes generate intrinsic contractile forces that engage mechanotransduction signalling [42]. Many disease‐associated *LMNA* mutations are known to disrupt lamin assembly and nuclear architecture and are therefore proposed to impair mechanotransduction [41]. This may limit the ability of the nucleus to sense and respond to mechanical forces. Here, the serine to proline substitution at p.143 introduces a structurally disruptive kink into the alpha‐helical rod domain close to the linker region, which is expected to perturb the normal protein–protein interactions at p.146, which are crucial for lateral assembly of lamin filaments and the formation of stable, organised nuclear lamina [43]. This structural defect may impair the efficient transmission of cytoskeletal forces to chromatin, blunting the activation of mechanosensitive transcription factors such as GATA4. Given that *NPPA* and *NPPB* are regulated through GATA4 [12, 25, 29, 44], this mechanism could contribute to the observed baseline downregulation of *NPPA*, *NPPB* and proANP in DCM cardiomyocytes. However, this mechanism remains speculative and warrants experimental validation.

We have previously reported that hiPSC cardiomyocytes exposed to mechanical stretching represent an advantageous platform for studying cardiomyocyte hypertrophy [12]. Cardiomyocyte hypertrophy is a multifaceted phenomenon that involves alterations in gene expression and protein synthesis, with additional effects on key processes such as intracellular signalling, contractile function and energy metabolism [45, 46]. One of the hallmark responses to pathological hypertrophy is the activation of a stress‐associated transcriptional programme, marked by the robust upregulation of genes such as *NPPB*, *NPPA*, *ACTA1* and *MYH7*. In this study, mechanical stretching induced a robust hypertrophic response in control cardiomyocytes, as indicated by significant upregulation of *NPPB* and *MYH7*, confirming that the stretching model effectively activates mechanosensitive gene expression. In HCM cardiomyocytes, the *NPPB* response was more modest at 24 h compared to control cardiomyocytes but became pronounced at 72 h, while *MYH7* was upregulated only at 72 h, which could indicate a delayed activation of hypertrophic gene expression in HCM cardiomyocytes. To our knowledge, mechanical stretching has not previously been applied to HCM hiPSC‐cardiomyocytes. A previous study using neurohormonal hypertrophic stimuli has shown that HCM hiPSC‐cardiomyocytes are particularly responsive to endothelin‐1 (ET‐1), which induces concentration‐dependent increases in cell surface area and myofibrillar disarray, hallmark features of hypertrophy, whereas other stimuli such as angiotensin II, insulin‐like growth factor‐1 and phenylephrine elicit less robust effects. Although Tanaka et al. [47] did not investigate hypertrophic gene expression and used only a single time point for ET‐1 exposure, which prevents assessment of delayed responses relative to controls, the robust hypertrophic effects they reported in HCM hiPSC‐cardiomyocytes indicate altered responsiveness to hypertrophic signals, consistent with the delayed *MYH7* upregulation we observed under mechanical stretch.

Upregulation of *NPPA* is also well established as a marker of mechanical stress [12, 33]. However, in this study, DCM cardiomyocytes responded to mechanical stretching with downregulation of *NPPA* and a corresponding trend toward reduced proANP, suggesting a blunted or maladaptive transcriptional response to mechanical load. As noted earlier, this structural defect in lamin A/C likely impairs GATA4 activation, which is also essential for a complete hypertrophic gene expression response and may thus contribute to the observed downregulation of *NPPA* upon stretch. Furthermore, DCM hiPSC‐cardiomyocytes have been reported to exhibit pronounced myofibrillar disorganisation and abnormal mechanosensitive Ca^2+^ signalling in response to stretch [48, 49], highlighting that DCM patient–derived cardiomyocytes differ from control cardiomyocytes in multiple aspects of their response to mechanical load. Overall, these findings suggest that while control hiPSC cardiomyocytes activate a typical hypertrophy‐associated gene response to mechanical stretch, HCM and DCM cardiomyocytes exhibit disease‐specific alterations in hypertrophic gene expression, characterised by delayed activation in HCM and attenuated responses in DCM, highlighting altered and context‐dependent transcriptional adaptation to mechanical stress.

In this study, we extend our previous observation that *GAL*, *CSRP3* and *SLC16A9* are transcriptionally regulated by mechanical stretch in hiPSC‐derived cardiomyocytes [12] by examining their expression in patient‐derived cardiomyocytes. *GAL*, a regulator of cardiac lipid and glucose metabolism [50] was elevated at baseline in both cardiomyopathy patient–derived lines and upregulated in all lines after 24 h of stretch. At 72 h, upregulation persisted in HCM cardiomyocytes, showed a similar trend in DCM cells, but was no longer observed in control cardiomyocytes. Given that pathological hypertrophy is associated with a shift toward increased glucose utilisation [51] and exogenous galanin has been reported to support this metabolic change in hypertrophied myocardium [52], stretch‐induced *GAL* expression may reflect attempts to maintain metabolic efficiency under mechanical load. However, whether *GAL* contributes to metabolic remodelling in genetic cardiomyopathies remains to be determined. *CSRP3*, a cytoskeletal protein involved in sarcomeric integrity and stretch sensing and previously reported to be upregulated as a compensatory response to cardiac injury [53, 54, 55], exhibited genotype‐dependent transcriptional responses to mechanical load in this study. These findings support its proposed role as a central component of the cardiac stretch–sensing machinery under biomechanical stress [53]. *CSRP3* has also been implicated in cardiomyocyte metabolism, with deficiency impairing lactate and ketone utilisation, indicating a reduced capacity to generate energy from alternative substrates under increased workload [56]. Together, these observations underscore the need for further studies on the role of *CSRP3* in structural and metabolic adaptations during cardiomyocyte stress. Finally, *SLC16A9*, an orphan monocarboxylate transporter [57, 58, 59], was consistently downregulated under stretch across all lines. Although no direct link between *SLC16A9* and cardiac diseases has been described, this uniform downregulation suggests that it may represent a robust transcriptional indicator of mechanical stress in hiPSC‐cardiomyocytes, independent of disease background.

In our previous work, the small molecule 3i‐1262 was shown to enhance cardiomyocyte maturation and exert anti‐hypertrophic effects: it enhanced metabolic and biosynthetic gene expression and reduced stretch‐induced hypertrophic signalling, including the upregulation of *NPPB* [30]. We then hypothesised that the compound may have similar or increased beneficial effects in genetic cardiomyopathy patient–derived cells. In the present study, we confirmed that 3i‐1262 reduced stretch‐induced *NPPB* upregulation in control cardiomyocytes; however, no comparable effect was observed in patient‐derived cardiomyocytes. Additionally, exposure to 3i‐1262 for 24 or 72 h did not affect cTnT protein levels in control cardiomyocytes, consistent with our previous Western blot findings [30]. Moreover, while mechanical stretch downregulated *SLC16A9*, 3i‐1262 counteracted its expression at 24 h in both control and HCM cardiomyocytes; however, this effect was no longer apparent at 72 h, suggesting that prolonged stretch exerts a more substantial regulatory influence on *SLC16A9* than the compound at this later time point. Of note, we did not add fresh compound during the 72‐h experiment, and compound degradation in the medium or cellular metabolism may have diminished its effects during prolonged treatment. Overall, these findings highlight genotype‐dependent variability in cardiomyocyte gene expression and support the use of patient‐specific or disease‐relevant cardiomyocytes for investigating cardiac pathophysiology and screening therapeutic interventions in a precision medicine context.

This study has several limitations. First, it investigated only one HCM‐associated mutation, one DCM‐associated mutation and one patient‐derived hiPSC line per genotype. As a result, the findings may not fully reflect the diverse genetic and phenotypic variations of HCM and DCM. Additionally, hiPSC‐derived cardiomyocytes exhibit an immature phenotype compared to adult cardiomyocytes, which may limit the direct translation of these findings. Furthermore, this study did not include functional assays, mechanistic validation or isogenic control lines. Future research that includes these approaches will enable genotype‐specific comparisons and clarify molecular and phenotypic disease mechanisms.

Despite these limitations, hiPSC‐derived cardiomyocytes represent a unique platform for cardiac research, enabling disease modelling and drug screening with human cardiomyocytes. Although their relatively immature phenotype remains a limitation [60], strategies such as co‐culture with non‐myocyte cell types, chemical stimulation and three‐dimensional culture systems have been used to promote maturation [60, 61, 62, 63]. Despite their immaturity, recent reports, including the present study, describe their robust hypertrophic responses and highlight them as a gold‐standard in vitro model for cardiac hypertrophy. Looking forward, advanced co‐culture and cardiac microtissue models incorporating multiple cardiac cell types are expected to more faithfully recapitulate the physiological cardiac environment, allowing the investigation of intercellular communication and its contribution to disease pathophysiology.

In summary, our study demonstrates that cardiomyocyte genotype influences both basal gene expression and responses to mechanical stretching. HCM and DCM hiPSC‐cardiomyocytes differ from control cardiomyocytes at baseline and respond to stretch in a disease‐specific manner. To our knowledge, this is the first report suggesting delayed hypertrophic activation in HCM, consistent with a blunted response in DCM. In control cardiomyocytes, 3i‐1262 attenuated stretch‐induced hypertrophic and metabolism‐related gene expression responses, whereas no clear effects were observed in HCM and DCM cells, highlighting that pharmacological modulation depends on genetic background. These findings underscore the utility of patient‐derived hiPSC‐cardiomyocytes as a platform for investigating disease mechanisms and for evaluating genotype‐specific therapeutic strategies and drug responses.

## Funding

This work was supported by Suomen Kulttuurirahasto, Sydäntutkimussäätiö, Research Council of Finland (2666621, 321564, 328909) and Sigrid Juséliuksen Säätiö.

## Conflicts of Interest

S.M.K., H.R. and M.J.V. are inventors in a patent WO2018055235—Isoxazole‐Amides for Treating Cardiac Diseases.

## Supporting information


**Table S1:** The primers used for DNA sequencing.
**Table S2:** TaqMan assays used in qPCR experiments.

**Figure S1:** DNA sequencing. The presence of the Gln1061X (A) and p.S143P (B) in the HCM and DCM hiPSCs, respectively, was confirmed by DNA sequencing.

**Figure S2:** Representative Western blots of cTnT (35 kDa) under basal and after 24 or 72 h of mechanical stretching, with or without treatment with the GATA4‐targeted compound 3i‐1262. Corresponding total‐protein membranes are provided in Supplementary Information Figure S4, where each membrane is labelled with the same running number as in this figure (e.g., membrane #1 corresponds to total protein #1). CTRL, control hiPSC‐cardiomyocytes; HCM, HCM hiPSC‐cardiomyocytes; DCM, DCM hiPSC‐cardiomyocytes; ET‐1, endothelin‐1, which was used as a positive control. The ‘+’ sign denotes the positive control: control hiPSC–derived cardiomyocytes from an independent differentiation treated with 100 nM endothelin‐1 for 24 h.

**Figure S3:** Representative Western blots of α‐MHC (224 kDa), β‐MHC (223 kDa), pro‐atrial natriuretic peptide (proANP; 18 kDa) and pro‐B‐type natriuretic peptide (proBNP; 15 kDa) under (A) basal conditions and after 24 h of mechanical stretching and (B) after 72 h of mechanical stretching. Corresponding total protein membranes are provided in Supplementary Information Figure S5, where each membrane is labelled with the same running number as in this figure (e.g., membrane #20 corresponds to total protein #20). CTRL, control hiPSC‐cardiomyocytes; HCM, HCM patient–derived hiPSC‐cardiomyocytes; DCM, DCM patient–derived hiPSC‐cardiomyocytes; ET‐1, endothelin‐1, which was used as a positive control. The ‘+’ sign denotes the positive control: control hiPSC–derived cardiomyocytes from an independent differentiation treated with 100 nM endothelin‐1 for 24 h.

**Figure S4:** Total protein under basal conditions and after 24 or 72 h of mechanical stretching, with or without treatment with the GATA4‐targeted compound 3i‐1262. CTRL, control hiPSC‐cardiomyocytes; HCM, HCM patient–derived hiPSC‐cardiomyocytes; DCM, DCM patient–derived hiPSC‐cardiomyocytes; ET‐1, endothelin‐1, which was used as a positive control. The ‘+’ sign denotes the positive control: control hiPSC–derived cardiomyocytes from an independent differentiation treated with 100 nM endothelin‐1 for 24 h.

**Figure S5:** Total protein under basal conditions and after 24 h (A) or 72 h (B) of mechanical stretching. CTRL, control hiPSC‐cardiomyocytes; HCM, HCM patient–derived hiPSC‐cardiomyocytes; DCM, DCM patient–derived hiPSC‐cardiomyocytes; ET‐1, endothelin‐1, which was used as a positive control. The “+” sign denotes the positive control: control hiPSC–derived cardiomyocytes from an independent differentiation treated with 100 nM endothelin‐1 for 24 h.

**Figure S6:** Relative expression of proANP, α‐MHC, β‐MHC and cTnT after 72 h of stretch in hiPSC‐cardiomyocytes. Results are presented as mean + SEM, and normalised to the respective, unstretched sample (*n* = 2–5). CTRL, control hiPSC‐cardiomyocytes; HCM, HCM patient–derived hiPSC‐cardiomyocytes; DCM, DCM patient–derived hiPSC‐cardiomyocytes. See Supplementary Information Figures S3B and S5B for the original Western blots and total protein images, respectively.

**Figure S7:** Relative expression of cTnT in control, HCM and DCM hiPSC‐cardiomyocytes following treatment with 30 μM 3i‐1262 for 24 or 72 h under unstretched conditions (A) and in combination with mechanical stretch (B). Results are presented as mean + SEM. In panel (A), results are normalised to the respective unstretched control, whereas in panel (B), the results are normalised to the respective stretched sample (*n* = 3). CTRL, control hiPSC‐cardiomyocytes; HCM, HCM patient–derived hiPSC‐cardiomyocytes; DCM, DCM patient–derived hiPSC‐cardiomyocytes. See Supplementary Information Figures S2 and S4 for the original Western blots and total protein image, respectively.

## Data Availability

The data that support the findings of this study are available from the corresponding author upon reasonable request.
