# Supplementary material for: Genotype‐Dependent Effects of Mechanical Stretch and GATA4‐Targeted Compound 3i‐1262 in Cardiomyopathy Patient–Derived hiPSC‐Cardiomyocytes
Source: Basic Clin Pharmacol Toxicol. 2026 Aug 18;139(3):e70284. doi: 10.1111/bcpt.70284 (PMC13484559; doi:10.1111/bcpt.70284)
Supplement: Supplementary file 1 [file BCPT-139-0-s003.pdf]

Supporting information

Genotype-dependent effects of mechanical stretch and GATA4-targeted compound 3i-1262 in cardiomyopathy patient-derived hiPSC-cardiomyocytes

Saana Pohjavaara[1], Sini M. Kinnunen[1], Heikki Ruskoaho[1], Katriina Aalto-Setälä[2,3], Mika J. Välimäki[1], Virpi Talman * [1, 4]

* Corresponding author, email: virpi.talman@utu.fi

[1] Drug Research Program and Division of Pharmacology and Pharmacotherapy, Faculty of Pharmacy, University of Helsinki, Helsinki, Finland
[2] Faculty of Medicine and Health Technology and BioMediTech Institute, Tampere University, Tampere, Finland
[3] Heart Hospital, Tampere University Hospital, Tampere, Finland
[4] Research Centre for Integrative Physiology and Pharmacology, Institute of Biomedicine, Faculty of Medicine, University of Turku, Turku, Finland

Table of contents

Supplementary methods	p. 2
Supplementary tables	p. 4
Supplementary figures	p. 5
References	p. 10

Supplementary methods

DNA Sequencing

PCR fragments of 324 bp and 323 bp around the p.S143P and the Gln1061X mutations, respectively, were amplified using PCR reaction that was conducted according to Phusion™ High-Fidelty DNA Polymerase kit (Thermo Scientific) and carried out in a 20 µL mixture containing 1X Phusion HF Buffer, 10 mM dNTPs, 0.5 µM both forward and reverse primers (Supplementary Table 1), 3 ng/µl template DNA and Phusion Polymerase. A thermocycler (Bio-Rad) was utilised to perform the amplification program. For the DCM hiPSC line, the protocol consisted of an initial denaturation at 98°C for 30 seconds, followed by 34 cycles of 98°C for 10 seconds, 63°C for 12 seconds, and 72°C for 14 seconds, with a final extension at 72°C for 10 minutes. For the HCM hiPSC line, the same program was used, except the annealing temperature was adjusted to 64.7°C. The gel-electrophoresis was run for the PCR products on a 1.3 % agarose gel and purified according to GeneJET Gel Extraction Kit (Thermo Scientific). The samples were sent for sequencing at eurofins Genomics.

Cell culture and differentiation

hiPSCs were maintained in Essential 8™ medium in feeder-free cultures on Matrigel-coated plates in standard conditions (+37 °C, in a humidified atmosphere of 5% CO_2) and passaged 1:15 approximately every four days using Versene. Upon passaging, ROCK inhibitor Y-27632 (Tocris; 10 µM) was added for 24 h. Differentiation into hiPSC-cardiomyocytes was initiated with 6 – 8 µM CHIR99021 (Tocris) in RPMI1640 medium supplemented with B-27 Minus Insulin (RB-) at 70–80% confluency (Day 0) [1]. On day 1 or day 2, cells were fed with fresh RB- (without CHIR99021), and on day 3 with RB- containing the Wnt inhibitor Wnt-C59 (Tocris) at 2.5 µM. Fresh RB- was changed on days 5, 7, and 9. To purify the culture from non-cardiomyocytes, metabolic selection was performed by culturing the cells in glucose-free RPMI medium supplemented with B-27 on days 11 and 13. From day 15 onwards, cardiomyocytes were fed twice a week with RPMI1640 medium supplemented with B-27 (RB+). The cells were dissociated on day 15–18 with TrypLE™ Select dissociation agent according to the manufacturer's instructions and seeded at 750.000 – 800.000 cells per well in RB+ with 10% heat-inactivated foetal bovine serum and ROCK inhibitor Y-27632 on flexible-bottom collagen I-coated 6-well BioFlex® culture plates (Flexcell International Corporation, Hillsborough, NC, USA) with additional thin-layer Matrigel coating. After 48h, the medium was changed to fresh RB+, and the cells were cultured until the initiation of cyclic mechanical stretching on days 29–41. Before initiating the experiments, cardiomyocyte cultures were observed under the microscope to visually confirm spontaneous beating. Experiments were only conducted if beating was clearly observed.

RNA isolation, cDNA synthesis, quantitative PCR

Total RNA was isolated with NucleoSpin RNA kit (Macherey–Nagel, Düren, Germany) according to the manufacturer's instructions. RNA concentration and quality were analysed with a NanoDrop Microvolume Spectrophotometer (Thermo Fisher Scientific, Waltham, MA, USA). Then, 115–1379 ng of RNA was reverse-transcribed to single-stranded cDNA in 10 – 20 µl reactions with Transcriptor First Strand cDNA Synthesis Kit (Roche, Basel, Switzerland) using random hexamer primers and MJ Mini Personal thermal cycler (Bio-Rad, Hercules, CA, USA). Both the cardiomyopathy cell line and its matched control were reverse-transcribed using equal amounts of RNA within each biological replicate (n = 1).

cDNA was diluted 1:10 in nuclease-free H2O and stored at -20°C. Commercial TaqMan® Gene Expression Assays from Thermo Fisher Scientific were used (Supplementary table 2) with the LightCycler® 480 ProbesMaster reagent (Roche), following the manufacturer's instructions. Reactions of 10 µl total volume, including 4.5 µl of diluted cDNA, were analysed in a white 384-well LightCycler® 480 plate and analysed using the LightCycler® 480 Real-Time PCR System (Roche). Each reaction was

performed in triplicate. Grubbs' test, with a significance level of 0.05, was employed to identify outliers within the technical replicates, and any identified outliers were excluded from further analysis. The relative gene expression was analysed using the 2−ΔΔCt method, in which the mean Ct value of *ACTB* and *18S rRNA* was used as an endogenous control [2].

Western blot

To enhance the detection of pro-B-type natriuretic peptide (proBNP), Brefeldin A (Invitrogen) was added to the cells three hours before cell lysis. After 24 or 72 hours of stretching, cells were lysed with 1% sodium dodecyl sulphate in 50 mM Tris-HCl (pH 7.5). Pierce™ BCA Protein Assay -kit (Thermo Scientific) was used to determine the total protein concentrations of the samples. Equal amounts of protein (15 µg or 20 µg of total protein) were loaded into 10% Mini-PROTEAN® TGX Stain-Free™ Protein gels (Bio-Rad). Along with the samples of interest, a sample from endothelin-1 (ET-1)-treated cells (100 nM) was included as a positive control [3]. The gels were exposed to UV light for 5 min to activate the reaction between trihalo compounds and proteins. Proteins were then transferred to a nitrocellulose membrane by Trans-Blot® Turbo™ transfer system (Bio-Rad). Non-specific binding was blocked with 5% non-fat dry milk in Tris-buffered saline with 0.1 % Tween 20 (TTBS) for 1 h at room temperature. The membranes were incubated overnight at 4°C in 5% milk-TTBS with primary antibodies against cardiac troponin T (cTnT;Abcam, ab45932; 1:1000), proBNP (Abcam, ab13115; 1:1000), α-myosin heavy chain (ThermoFisher Scientific, MA5-24631; 1:1000), β-myosin heavy chain (Santa Cruz, sc-53090; 1:200) and pro-atrial natriuretic peptide (proANP) (ThermoFisher Scientific, MA5-51086; 1:2500). The membranes were incubated for one hour in 1:2000 HRP-conjugated secondary antibody solutions (anti-rabbit (Cell Signaling Technology, 7074S); anti-mouse (Cell Signaling Technology, 7076). cTnT, α and β -myosin heavy chain bands were detected with SuperSignalTM West Pico PLUS (Thermo Scientific) ECL substrate, and proANP and proBNP bands were detected with SuperSignalTM West Femto Maximum Sensitivity Substrate (Thermo Scientific) using a ChemiDocTM MP Imaging System (Bio-Rad). Optical densities of the bands were quantified using the Fiji ImageJ 1.53 software. The densities of the protein bands were normalised to the total protein of the sample.

Supplementary tables

Supplementary table 1. The primers used for DNA sequencing.

Gene	Exon	Size (bp)	Primer sequence
MYBPC3	Exon 29	324	Frw: CTGGCCTGATCTGAGGTACCAG<br>Rev: GAAGGGAAACAAGGGGGCTCA
LMNA	Exon 5	323	Frw: CTAATGCAAGGATGCCCTCTC<br>Rev: GTGTACATGTGTTAGGTGGGGC

Supplementary table 2. TaqMan assays used in qPCR experiments.

Gene	Function	TaqMan® Gene Expression Assay ID
ACTB	Housekeeping gene	4352935E
18S rRNA	Housekeeping gene	4352930E
NPPB	Hypertrophy marker	Hs01057466_g1
NPPA	Hypertrophy marker	Hs00383230_g1
MYH6	Sarcomeric gene	Hs01101425_m1
MYH7	Sarcomeric gene	Hs01110632_m1
ACTC1	Sarcomeric gene	Hs01109515_m1
TNNT2	Sarcomeric gene	Hs00943911_m1
MYBPC3	Sarcomeric gene	Hs00165232_m1
ACTN2	Sarcomeric gene	Hs00153809_m1
ACTA1	Sarcomeric gene	Hs00559403_m1
CSRP3	Sarcomeric gene	Hs00185787_m1
GATA4	Cardiac transcription factor	Hs00171403_m1
GAL	Metabolic-related gene	Hs00544355_m1
SLC16A9	Metabolic-related gene	Hs00415854_m1

Supplementary figures

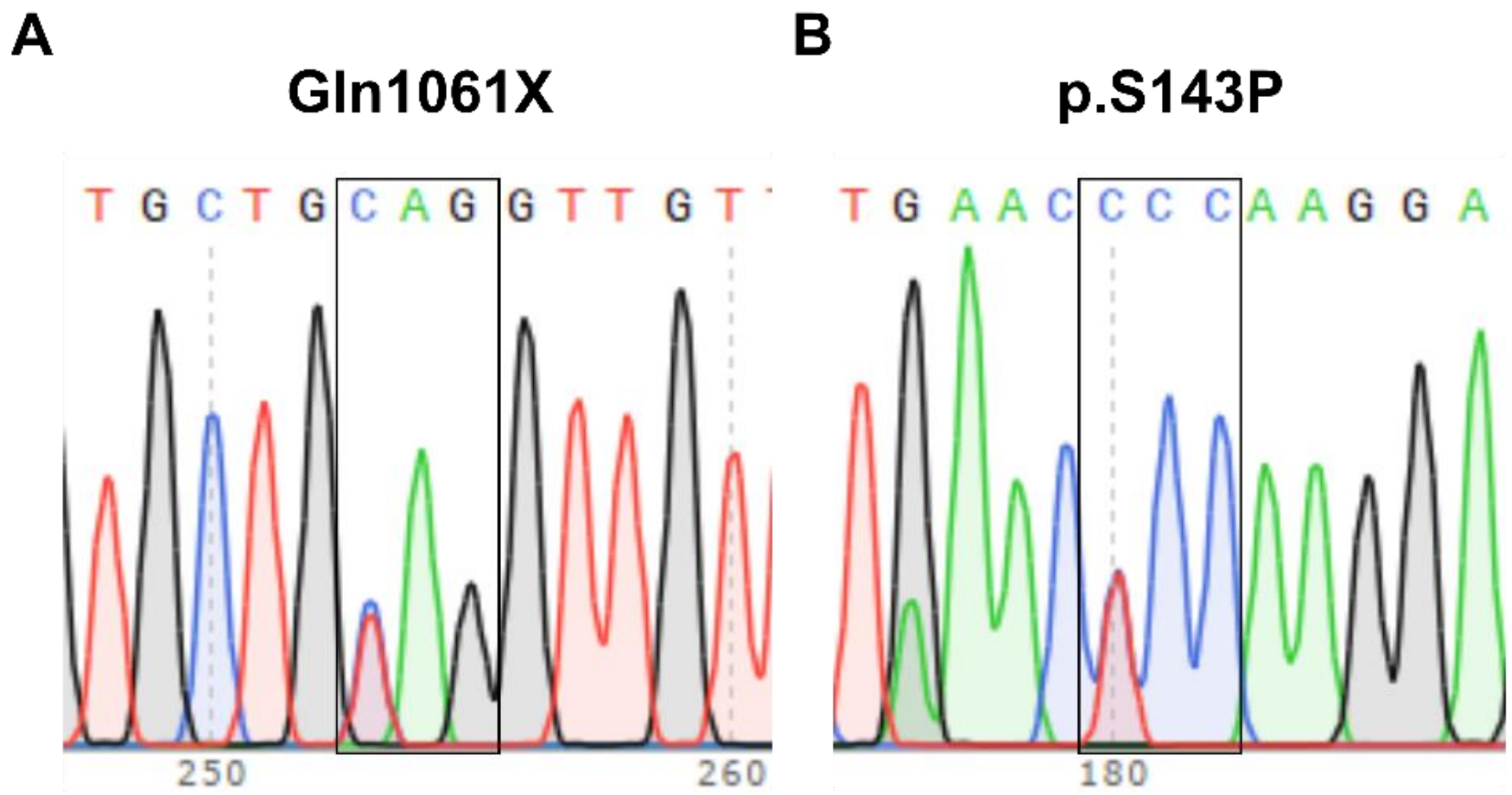

Supplementary figure 1. DNA sequencing. The presence of the Gln1061X (A) and p.S143P (B) in the HCM and DCM hiPSCs, respectively, was confirmed by DNA sequencing.

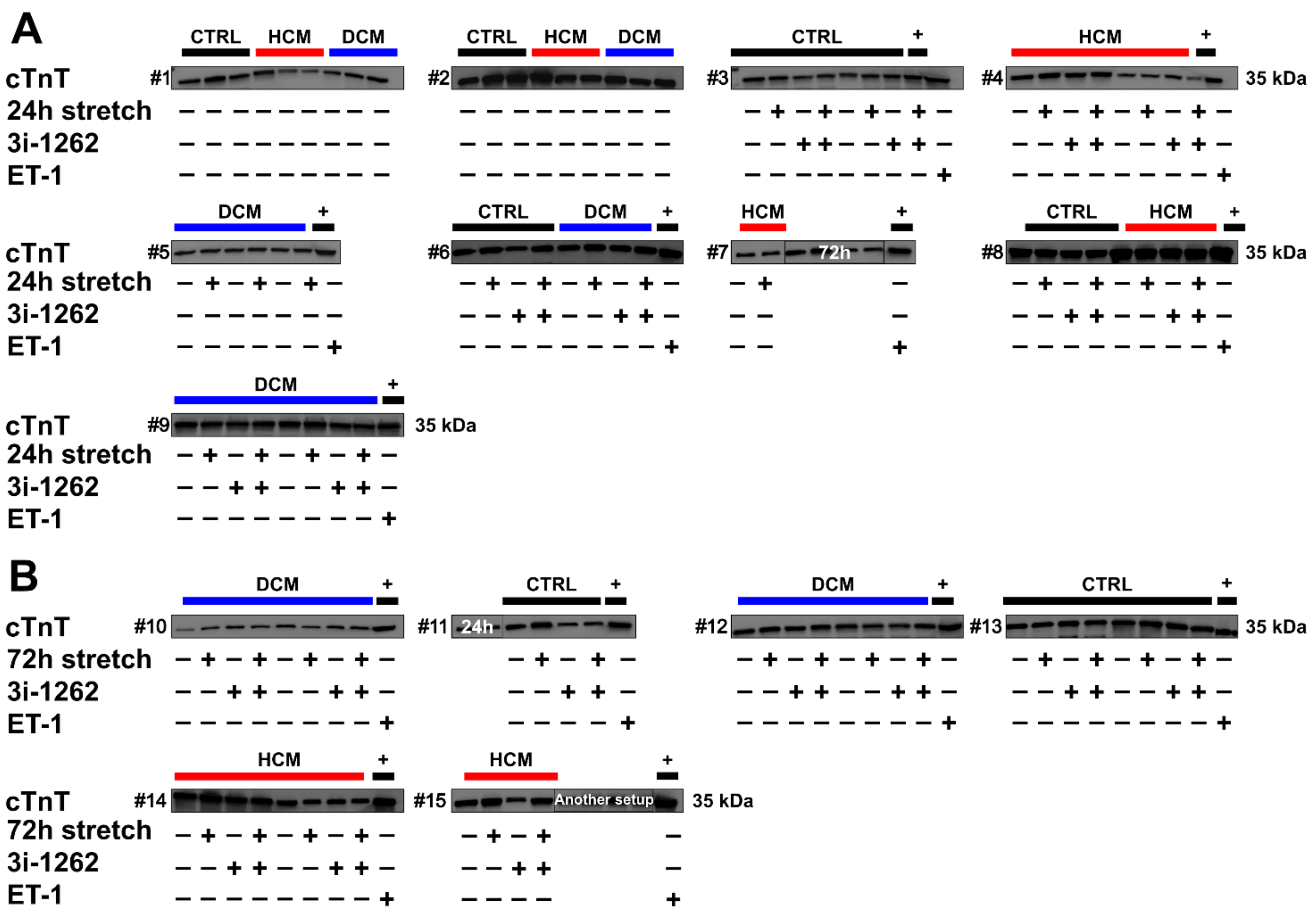

Supplementary figure 2. Representative Western blots of cardiac troponin T (cTnT; 35 kDa) under basal and after 24 or 72 hours of mechanical stretching, with or without treatment with the GATA4-targeted compound 3i-1262. Corresponding total-protein membranes are provided in Supplementary Fig. 4, where each membrane is labelled with the same running number as in this figure (e.g., membrane #1 corresponds to total protein #1CTRL, control hiPSC-cardiomyocytes; HCM, HCM hiPSC-cardiomyocytes; DCM, DCM hiPSC-cardiomyocytes; ET-1, endothelin-1, which was used as a positive control. The “+” sign denotes the positive control: control hiPSC-derived cardiomyocytes from an independent differentiation treated with 100 nM endothelin-1 for 24 hours.

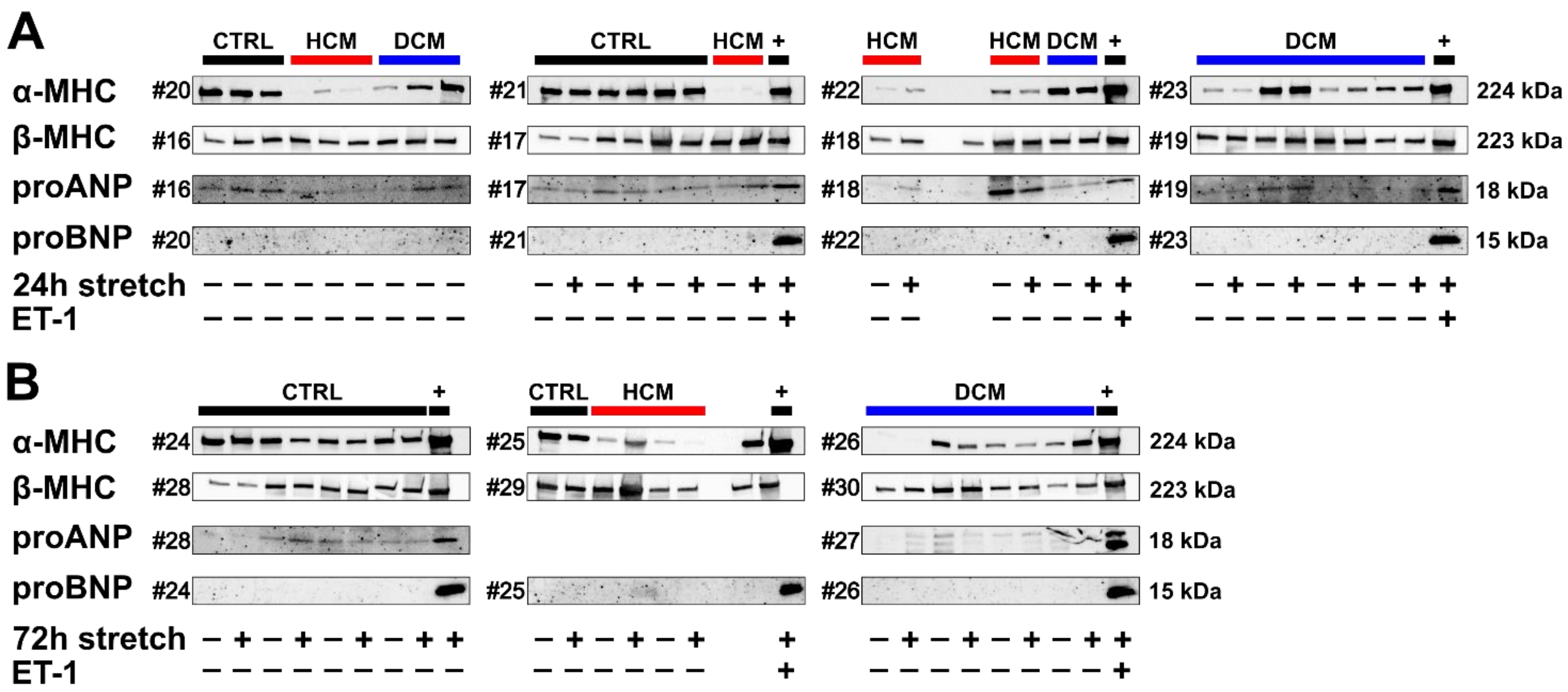

Supplementary figure 3. Representative Western blots of α-myosin heavy chain (α-MHC; 224 kDa), β-myosin heavy chain (β-MHC; 223 kDa), pro-atrial natriuretic peptide (proANP; 18 kDa), and pro-B-type natriuretic peptide (proBNP; 15 kDa) under (A) basal conditions and after 24 h of mechanical stretching and (B) after 72 h of mechanical stretching. Corresponding total protein membranes are provided in Supplementary Fig. 5, where each membrane is labelled with the same running number as in this figure (e.g., membrane #20 corresponds to total protein #20). CTRL, control hiPSC-cardiomyocytes; HCM, HCM patient-derived hiPSC-cardiomyocytes; DCM, DCM patient-derived hiPSC-cardiomyocytes; ET-1, endothelin-1, which was used as a positive control. The "+" sign denotes the positive control: control hiPSC-derived cardiomyocytes from an independent differentiation treated with 100 nM endothelin-1 for 24 hours.

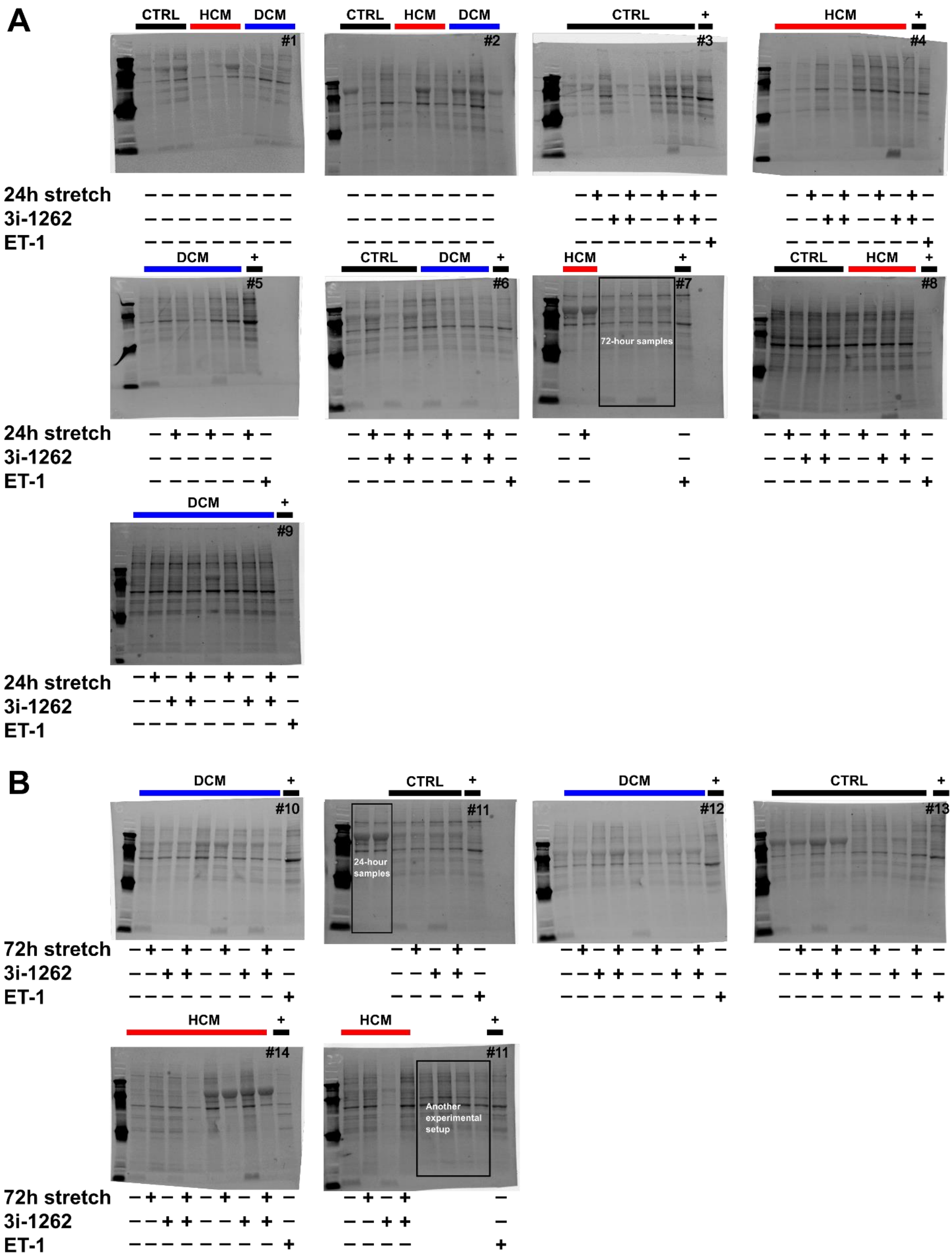

Supplementary figure 4. Total protein under basal conditions and after 24 or 72 hours of mechanical stretching, with or without treatment with the GATA4-targeted compound 3i-1262. CTRL, control hiPSC-cardiomyocytes; HCM, HCM patient-derived hiPSC-cardiomyocytes; DCM, DCM patient-derived hiPSC-cardiomyocytes; ET-1, endothelin-1, which was used as a positive control. The “+” sign denotes the positive control: control hiPSC-derived cardiomyocytes from an independent differentiation treated with 100 nM endothelin-1 for 24 hours.

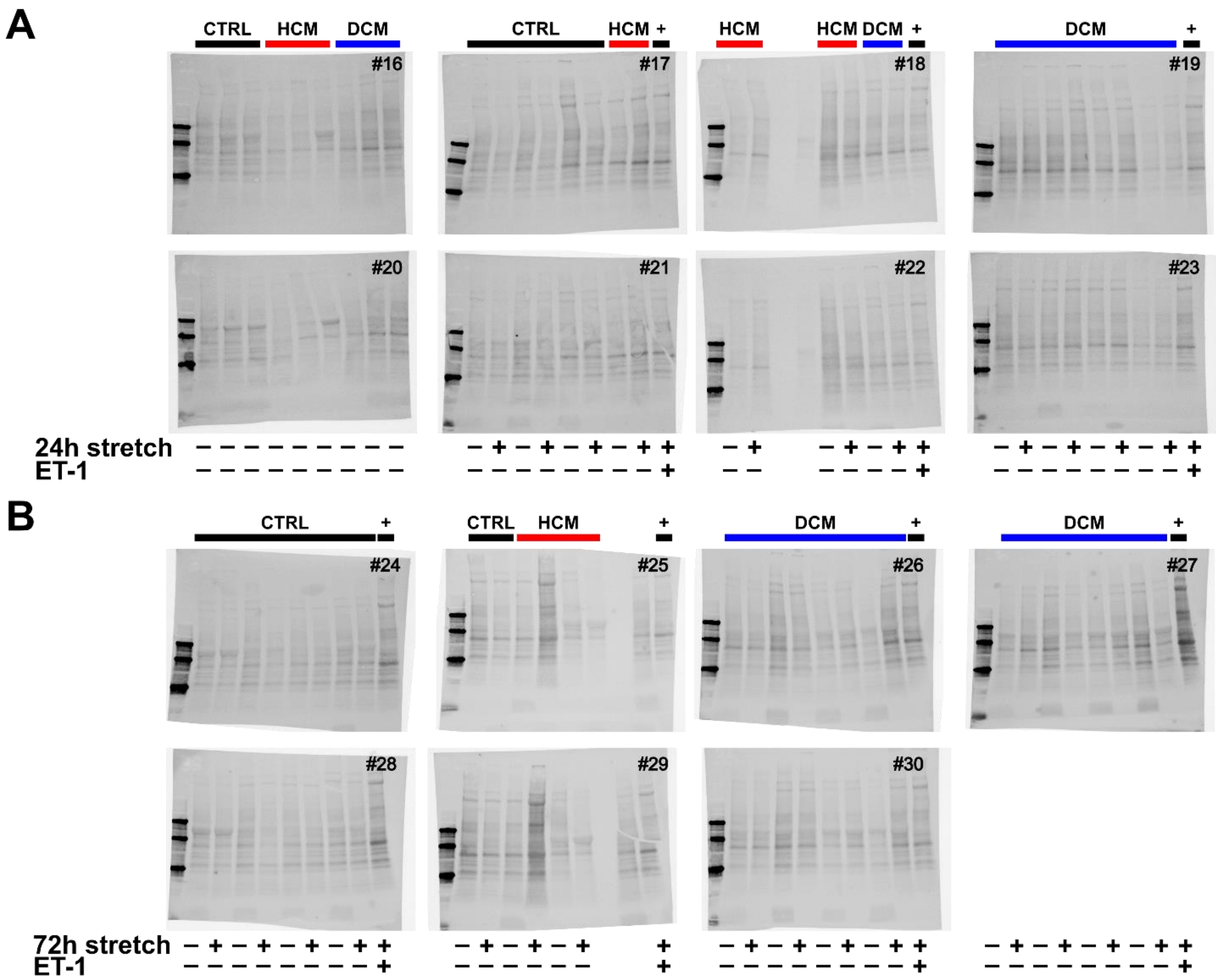

Supplementary figure 5. Total protein under basal conditions and after 24h (A) or 72h (B) of mechanical stretching. CTRL, control hiPSC-cardiomyocytes; HCM, HCM patient-derived hiPSC-cardiomyocytes; DCM, DCM patient-derived hiPSC-cardiomyocytes; ET-1, endothelin-1, which was used as a positive control. The "+" sign denotes the positive control: control hiPSC-derived cardiomyocytes from an independent differentiation treated with 100 nM endothelin-1 for 24 hours.

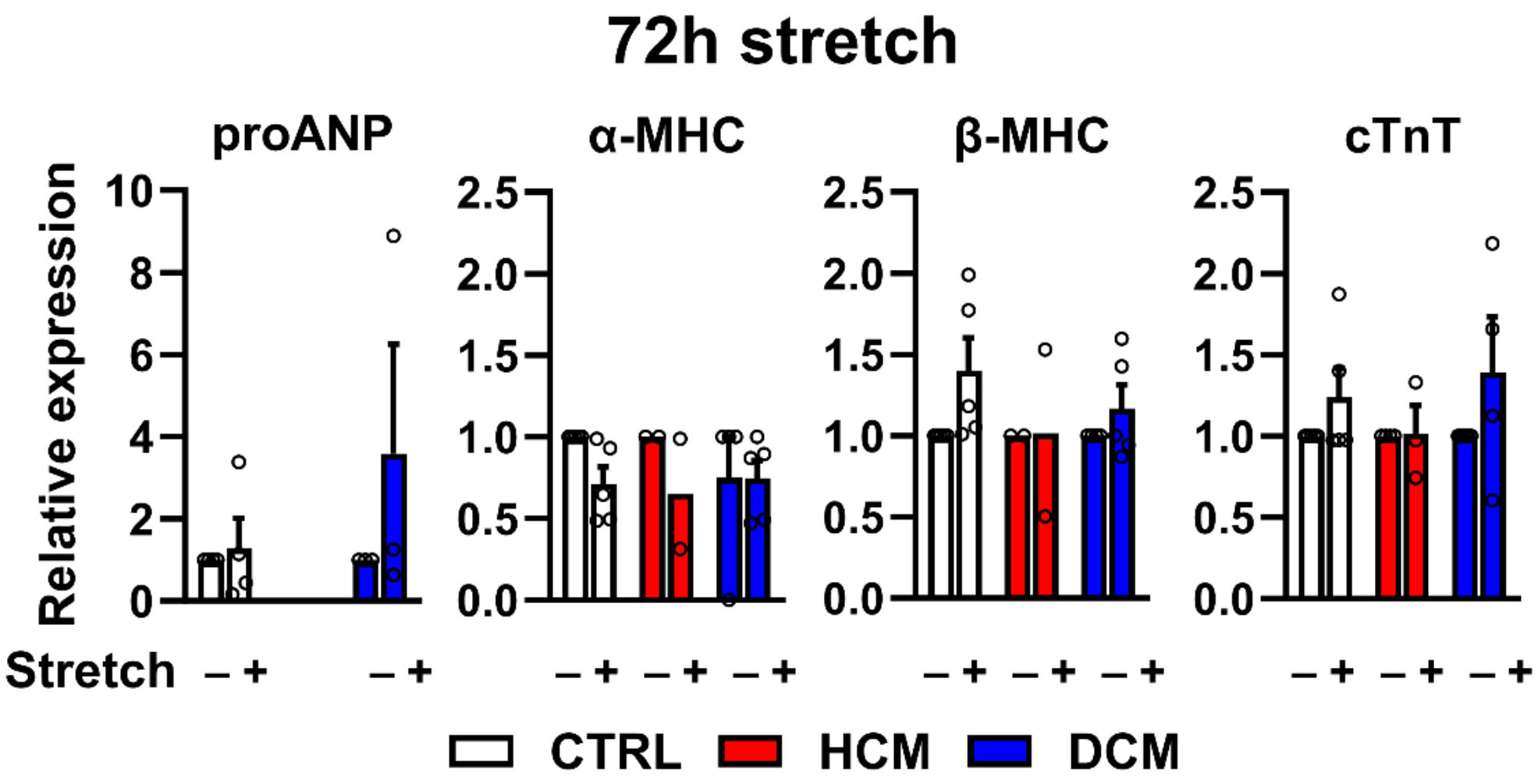

Supplementary figure 6. Relative expression of pro-atrial natriuretic peptide (proANP), α-myosin heavy chain (α-MHC), β-myosin heavy chain (β-MHC), and cardiac troponin T (cTnT) after 72 hours of stretch in hiPSC-cardiomyocytes. Results are presented as mean + SEM, and normalised to the respective, unstretched sample (n = 2–5). CTRL, control hiPSC-cardiomyocytes; HCM, HCM patient-derived hiPSC-cardiomyocytes; DCM, DCM patient-derived hiPSC-cardiomyocytes. See supplementary figure 3B and 5B for the original Western blots and total protein images, respectively.

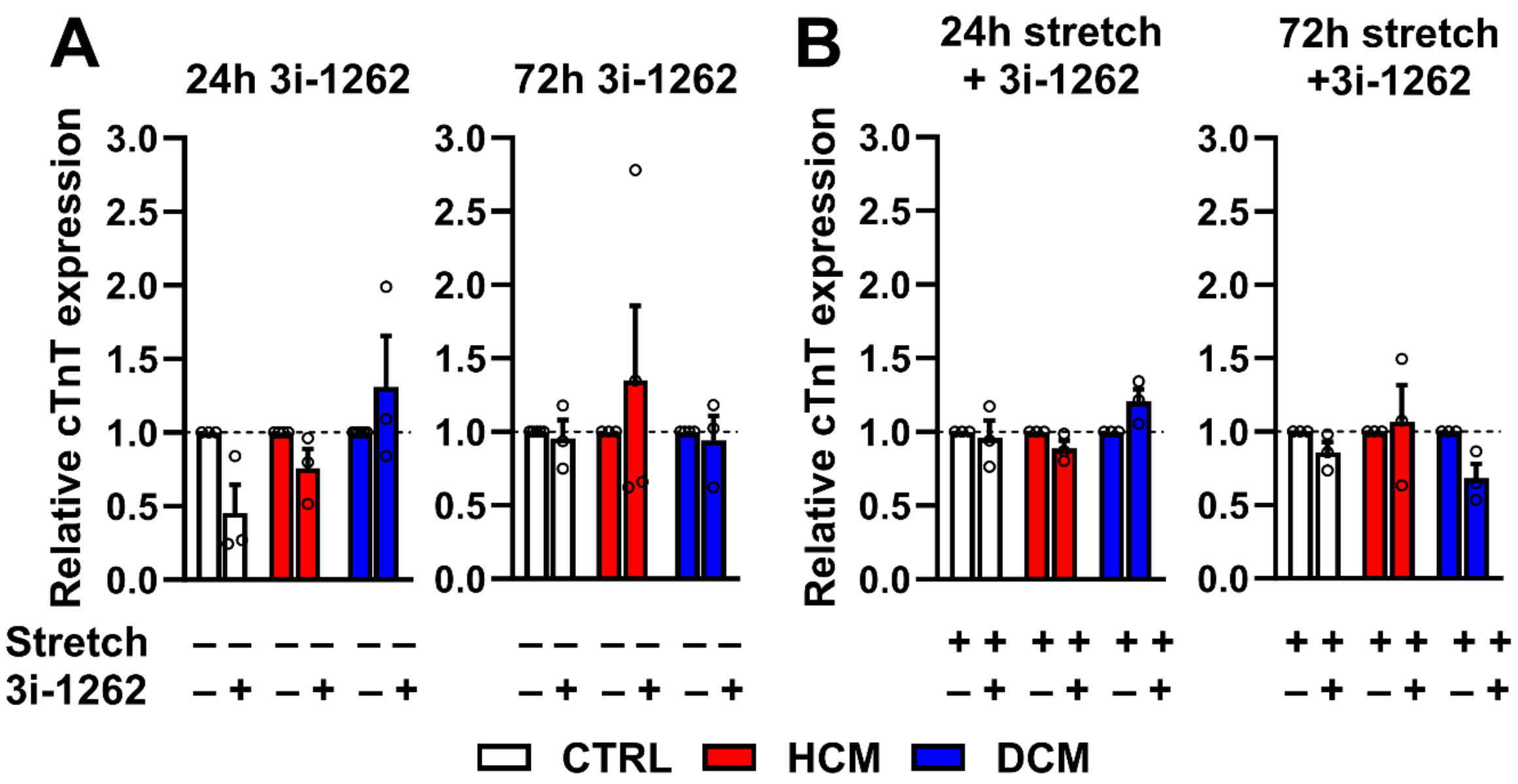

Supplementary figure 7. Relative expression of cardiac troponin T (cTnT) in control, HCM, and DCM hiPSC-cardiomyocytes following treatment with 30 µM 3i-1262 for 24 h or 72 h under unstretched conditions (A) and in combination with mechanical stretch (B). Results are presented as mean + SEM. In panel (A), results are normalised to the respective unstretched control, whereas in panel (B), the results are normalised to the respective stretched sample, (n = 3). CTRL, control hiPSC-cardiomyocytes; HCM, HCM patient-derived hiPSC-cardiomyocytes; DCM, DCM patient-derived hiPSC-cardiomyocytes. See supplementary figures 2 and 4 for the original Western blots and total protein image, respectively.

References

1. Burridge, P. W. *et al.* Chemically Defined Generation of Human Cardiomyocytes. *Nat. Methods* **11**, 855–860 (2014).
2. Pohjolainen, L., Ruskoaho, H. & Talman, V. Transcriptomics reveal stretched human pluripotent stem cell-derived cardiomyocytes as an advantageous hypertrophy model. *Journal of Molecular and Cellular Cardiology Plus* **2**, 1–14 (2022).
3. Pohjolainen, L. *et al.* Switching of hypertrophic signalling towards enhanced cardiomyocyte identity and maturity by a GATA4 - targeted compound. *Stem Cell Res. Ther.* **15**, 1–24 (2024).
